# Intervening on Trust in Science to Reduce Belief in COVID-19 Misinformation and Increase COVID-19 Preventive Behavioral Intentions: Randomized Controlled Trial

**DOI:** 10.2196/32425

**Published:** 2021-10-14

**Authors:** Jon Agley, Yunyu Xiao, Esi E Thompson, Xiwei Chen, Lilian Golzarri-Arroyo

**Affiliations:** 1 Prevention Insights, Department of Applied Health Science School of Public Health Bloomington Indiana University Bloomington Bloomington, IN United States; 2 Department of Population Health Sciences Weill Cornell Medicine New York, NY United States; 3 Indiana University Media School Indiana University Bloomington Bloomington, IN United States; 4 Biostatistics Consulting Center School of Public Health Bloomington Indiana University Bloomington Bloomington, IN United States

**Keywords:** infodemic, misinformation, trust in science, COVID-19, RCT, randomized controlled trial

## Abstract

**Background:**

Trust in science meaningfully contributes to our understanding of people’s belief in misinformation and their intentions to take actions to prevent COVID-19. However, no experimental research has sought to intervene on this variable to develop a scalable response to the COVID-19 infodemic.

**Objective:**

Our study examined whether brief exposure to an infographic about the scientific process might increase trust in science and thereby affect belief in misinformation and intention to take preventive actions for COVID-19.

**Methods:**

This two-arm, parallel-group, randomized controlled trial aimed to recruit a US representative sample of 1000 adults by age, race/ethnicity, and gender using the Prolific platform. Participants were randomly assigned to view either an intervention infographic about the scientific process or a control infographic. The intervention infographic was designed through a separate pilot study. Primary outcomes were trust in science, COVID-19 narrative belief profile, and COVID-19 preventive behavioral intentions. We also collected 12 covariates and incorporated them into all analyses. All outcomes were collected using web-based assessment.

**Results:**

From January 22, 2021 to January 24, 2021, 1017 participants completed the study. The intervention slightly improved trust in science (difference-in-difference 0.03, SE 0.01, t_1000_=2.16, *P*=.031). No direct intervention effect was observed on belief profile membership, but there was some evidence of an indirect intervention effect mediated by trust in science (adjusted odds ratio 1.06, SE 0.03, 95% CI 1.00-1.12, *z*=2.01, *P*=.045) on membership in the “scientific” profile compared with the others. No direct nor indirect effects on preventive behaviors were observed.

**Conclusions:**

Briefly viewing an infographic about science appeared to cause a small aggregate increase in trust in science, which may have, in turn, reduced the believability of COVID-19 misinformation. The effect sizes were small but commensurate with our 60-second, highly scalable intervention approach. Researchers should study the potential for truthful messaging about how science works to serve as misinformation inoculation and test how best to do so.

**Trial Registration:**

NCT04557241; https://clinicaltrials.gov/ct2/show/NCT04557241

**International Registered Report Identifier (IRRID):**

RR2-10.2196/24383

## Introduction

### Background

The COVID-19 pandemic has been accompanied by a substantive, pervasive outpouring of misinformation about the disease [1] that can be described as an infodemic [2]. Concerns about this infodemic were raised by members of the science community almost immediately, and steps were taken to develop a research agenda [3], as misinformation about COVID-19 has taken many forms and been amplified across numerous types of media [4-6]. Anecdotal stories about behaviors and consequences associated with COVID-19 misinformation can be readily identified (as we have done in small measure in our prior work [7,8]), and some scholars have documented negative outcomes of COVID-19 misinformation [9,10]. Though Greene and Murphy [11] recently found “surprisingly little” experimental research examining the effects of misinformation on behavior, their study found that even brief, single exposures to COVID-19 misinformation may nudge (have a small effect on) some behavioral intentions. Loomba et al [12] similarly found evidence for decreases in COVID-19 vaccination intention due to exposure to misinformation.

Unfortunately, far from tapering more than a year into the pandemic, the volume of misinformation has remained high; representatives of multiple organizations, including the World Health Organization and US Food and Drug Administration, recently warned that misinformation poses a global concern and may drive pandemic-related harms [13]. It is clearly incumbent on researchers to develop a thorough understanding of COVID-19 misinformation and to establish evidence-based mitigation tools.

### Belief in COVID-19 Misinformation Clusters Is Associated With Trust in Science

In May 2020, in response to growing concern about the COVID-19 infodemic, our team conducted one of the first studies of COVID-19 misinformation believability and the factors associated therewith [7]. We examined 5 brief narrative statements ranging from clearly false (eg, 5G transmission of COVID-19) to likely misinformed or improbable but not impossible (eg, purposeful laboratory development as a weapon) to a statement reflecting the scientific consensus at the time (eg, zoonotic origin). Using latent profile analysis, we identified 4 belief profiles into which it was possible to classify participants. Members of the largest profile (70.15% of the sample) reported high believability for a statement about the zoonotic origin and much lower believability for the misinformed statements. Members of the other, smaller profiles did not disbelieve the zoonotic statement, but tended to report higher believability for misinformation. In other words, findings suggested the existence of a large “scientific” or science-consistent group and multiple smaller groups that found misinformation believable to various degrees.

Then, we found that—controlling for race/ethnicity, gender, age, and education level—trust in science and scientists, a scale variable computed from 21 Likert-type questions of the Trust in Science and Scientists Inventory [14], was strongly associated with belief profile membership, with greater trust being associated with considerably higher odds of belonging to the “scientific profile.” The magnitude of adjusted odds for trust substantially exceeded that of other variables hypothesized to be associated with profile membership (political orientation and religious commitment) that were simultaneously analyzed [7].

Based on our findings and research described subsequently, we speculated that the strong association between COVID-19 narrative belief profile and trust in science might mean that (1) if a brief, inexpensive intervention could increase trust in science, it might possibly (2) affect individuals’ COVID-19 narrative belief profile membership. We also wondered whether this effect, mediated by belief profile, might (3) influence behavioral intentions to undertake COVID-19 preventive behaviors. Much of our rationale for these ideas is laid out in the published protocol for the present study [8]. Here, we present a brief explanation outlining why we have focused on trust and how this study fits among current COVID-19 misinformation interventions.

### Trust in Science May be an Effective Intervention Target for Misinformation Prevention

#### Theoretical Basis for Focusing on Trust

Trust is highly complex [14]. Often, “we know by trusting what others tell us” [15]. This is the case because there are many things about which we cannot produce our own knowledge, but there are often experts who *do* have that capability. Here, we posit that beliefs about COVID-19 are linked to rational epistemic trust, the idea that it is reasonable to believe statements made by experts. This might be expressed by the principle, “If [person] has good reasons to believe that [scientist] has good reasons to believe [a finding], then [person] has good reasons to believe that [finding]” [16].

Importantly, though, the prior formulation only pertains to claims about research findings, which make assertions about reality with varying degrees of certainty (eg, face mask use can reduce community transmission of COVID-19 [17]). *Recommendations* from scientists or experts, which may be *based on* scientific claims, instead suggest what people can do to achieve a specific outcome, and so they appeal to a different form of trust. For example, the manuscript reporting that face mask use could reduce community transmission also stated, “face mask use should be as nearly universal as possible” [17]. However, one can be logically consistent and believe both that face mask use can prevent community spread (eg, trust the findings of the research study) and that face mask use should not be universal (eg, not thinking that preventing community spread is important or that it is less important than other interests such as social identity [18]). An alternate formulation that pertains to trusting recommendations might be, “If [person] has good reasons to believe [scientist] has good reasons to believe that a certain action is in [person’s] interest, then [person] has good reasons to believe that they should perform that action” [19].

These concepts can help illustrate how the Trust in Science and Scientists Inventory [14] might be associated with COVID-19 preventive behavioral intentions or belief in COVID-19 misinformation. For example, the item “when scientists change their mind about a scientific idea, it diminishes my trust in their work” might reflect the conditions in which a person trusts a scientific finding, or the item “today’s scientists will sacrifice the well-being of others to advance their research” could inform our understanding of how a person perceives recommendations from a scientist.

#### Evidentiary Basis for Focusing on Trust

In addition to our own identification of associations between trust in science and belief in misinformation [7], studies conducted early in the pandemic found that willingness to abide by COVID-19 preventive guidelines was directly associated with trust in science and risk perception [20-22], the former of which also served as a mediator [22] or moderator [23] for other characteristics such as political conservatism. Trust in science has also been associated with intention to get vaccinated for COVID-19 [21,24]. However, we have not located any prior studies examining misinformation as a mediator in any such relationships.

### Extant Interventions to Reduce the Influence of COVID-19 (and Related) Misinformation

One prominent approach to addressing misinformation is debunking (eg, fact checking). Despite some initial concerns, fact checking appears unlikely to backfire [25], and a recent Dutch randomized controlled trial demonstrated that debunking messaging can reduce endorsement of myths about vaccines and influenza [26]. At the same time, fact checking still suffers from scalability issues and a variety of other nuanced concerns and effects [27]. For example, on its face, the time and effort needed to prepare and disseminate a specific piece of misinformation are typically less than the time and effort spent debunking it. Thus, fact checking is likely a useful but not sufficient response amid rapid proliferation of misinformation. Other issues also add complexity to debunking, such as the recent finding that social media users who reported correcting others about COVID-19 online were also more likely to endorse misperceptions about the disease [28].

A promising additional approach is prebunking (eg, inoculation) to confer resistance to the potential influence of misinformation before it is encountered [29-31]. Somewhat related are recent, robust studies suggesting that the likelihood of sharing fake news can be reduced by interventions to reduce inattention and encourage a focus on accuracy [32,33]. Further, in one study, active (online game) and passive (infographic) prebunking interventions targeting misinformation and fake news improved participants’ ability to identify misinformation about COVID-19, and the active condition also reduced willingness to share it [34].

### Study Aims and Hypotheses

#### Rationale

In our prior study of COVID-19 misinformation, around 70% of respondents were classified as belonging to the “scientific profile,” and classification therein was strongly associated with trust in science. Such an association is also supported both by theoretical and scientific literature. Separately, research on COVID-19 misinformation has suggested the value of scalable, universal prophylaxis that can support people in resisting the influence of misinformation. Therefore, our current study combines those ideas to examine an inoculation approach to COVID-19 misinformation using trust in science as a scalable intervention target.

In this preregistered, randomized controlled trial, we examined the effects of a brief prophylactic intervention (viewing a single infographic about the scientific process for at least 60 seconds). The study had 3 aims with corresponding hypotheses, which we copied verbatim from the study protocol [8] here for narrative clarity.

#### Aim 1

We aim to assess the effect of a brief informational infographic about the scientific process on trust in science. We hypothesize that exposure to such an intervention will have a moderate, positive effect on trust in science.

#### Aim 2

We aim to assess the effect of a brief informational infographic about the scientific process on the likelihood of believing scientifically implausible narratives about COVID-19. We hypothesize that exposure to such an intervention will have a small, negative effect on the likelihood of believing implausible narratives, as evidenced by profile membership, and that this will be partly mediated by trust in science.

#### Aim 3

We aim to assess the effect of a brief informational infographic about the scientific process on behavioral intentions to engage in recommended COVID-19 [nonpharmaceutical preventive behaviors (NPBs)]. We hypothesize that exposure to such an intervention will have a small, positive effect on behavioral intentions to engage in recommended COVID-19 NPBs that will be partly mediated by misinformation profile membership.

## Methods

### Study Design and Participants

This study of COVID-19 misinformation prophylaxis was a single-stage, two-arm, parallel-group, randomized superiority trial with a 1:1 allocation ratio. Participants were a US-based nationally representative population sample by age, sex, race, and ethnicity recruited using the online data collection platform Prolific [35]. Participants were eligible for this study if they were aged 18 years or older and were selected by Prolific to be part of the nationally representative sample. Prior to randomization, evidence-based quality control mechanisms to manage virtual private network usage, automated responses, dishonest respondents, and inattentive respondents were implemented [36], and participants were considered ineligible if they failed any of these steps. Replacements were drawn in a manner that preserved the representative nature of the sample. All participants provided digitally signed informed consent according to the protocol approved by the Indiana University Institutional Review Board. This study was preregistered with Clinicaltrials.gov (NCT04557241), and the protocol was published in full before any data collection [8].

### Randomization and Masking

**Figure 1:**
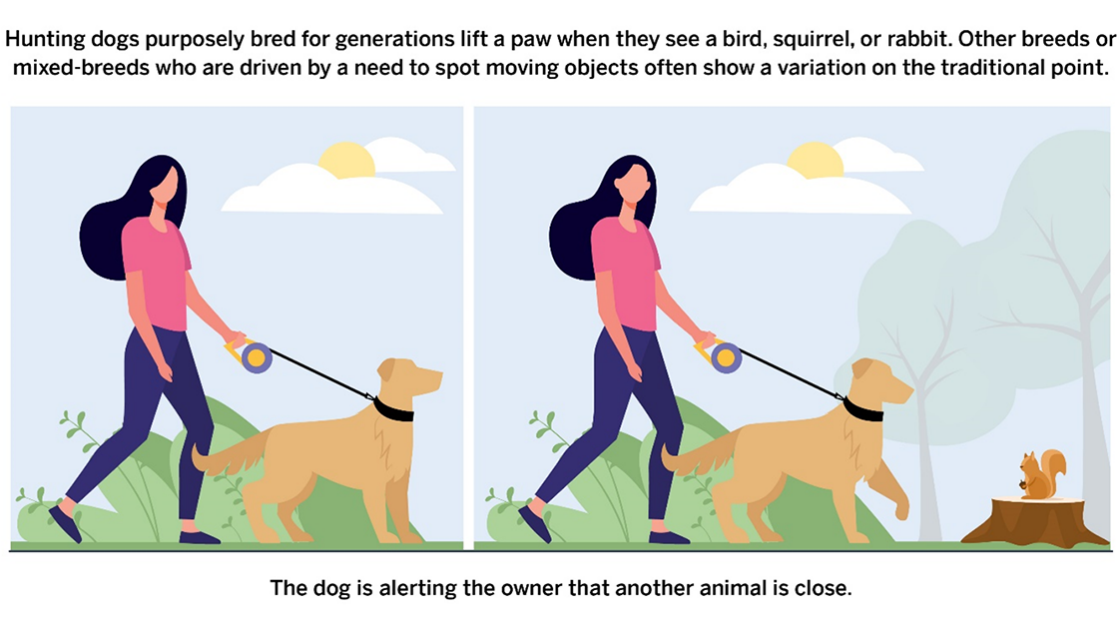
Control infographic.

**Figure 2:**
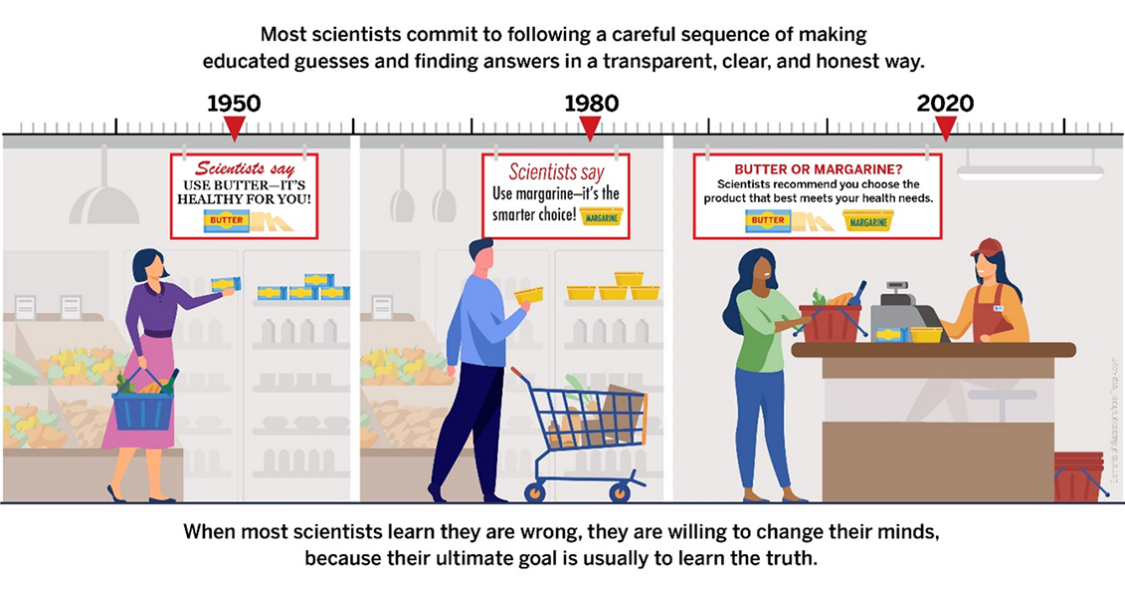
Intervention infographic.

After providing sociodemographic information and passing quality control checks, participants were randomly assigned to 1 of 2 study arms: (1) a control group that viewed an infographic about how hunting dogs point at targets (Figure 1) or (2) an intervention group that viewed an infographic about purchasing butter and margarine at the grocery store that was intended to highlight how scientific recommendations change along with newly available evidence (Figure 2). The same artist designed both infographics.

Enrollment was managed by Prolific, entirely independent of the study team. Enrolled subjects accessed a link to Qualtrics (QualtricsXM, Seattle, WA) to participate in the study. Eligible participants were randomized to study arms using the randomizer procedure in Qualtrics with a 1:1 allocation ratio, ensuring no involvement by study personnel. To prevent expectancy biases, study hypotheses and intentions were masked to participants. The summary statement indicated only that “we are interested in understanding how people perceive and think about messages and images.”

### Procedures

As prespecified [8], the intervention infographic (Figure 2) was iteratively developed using a multistage pilot procedure prior to study initiation. The results of that procedure, which included a randomized pilot comparison between 5 potential infographics, are described in a separate publication [37]. Participants who entered the Qualtrics survey completed sociodemographic items (see the Covariates section) and quality control checks, the latter of which are described in detail in a separate methodological paper [36].

Eligible participants who passed quality control checks were randomized (no indication of this was provided to participants) and then proceeded to the Trust in Science and Scientists Inventory [14]. Then, the program proceeded to display either the control or intervention infographic for the participant to view for a minimum of 60 seconds. To ensure maximum visibility of the infographic for participants on multiple platforms, the Lightbox script [38] was integrated into Qualtrics to allow participants to manually enlarge and reduce images. Following the intervention, participants were asked about the believability of 7 statements about COVID-19 and then were asked about 7 behavioral intentions based on recommendations by the US Centers for Disease Control and Prevention (CDC). These measures were described in detail in the protocol [8], and their use in this study is described in the Outcomes section. Finally, participants completed the Trust in Science Inventory a second time.

### Outcomes

This study had 3 primary prespecified outcome measures corresponding with 3 aims.

#### Aim One

Aim One investigated the effect of the intervention on participants’ trust in science and scientists. That construct was measured using the 21-item Trust in Science Inventory [14], which produces a composite score ranging from 1 (low trust) to 5 (high trust). Items in this inventory use Likert-type responses to statements like, “When scientists change their mind about a scientific idea, it diminishes my trust in their work,” and “Scientists will protect each other even when they are wrong.”

#### Aim Two

Aim Two investigated the effect of the intervention on participants’ classification into misinformation believability profiles [8]. To compute these profiles, participants were asked how believable they found 7 different statements about COVID-19, with responses ranging from 1 (extremely unbelievable) to 7 (extremely believable). Four of these statements were used in our prior research [7] and were derived from an early list of COVID-19 misinformation [5]:

The rollout of 5G cellphone networks caused the spread of COVID-19.

Bill Gates caused (or helped cause) the spread of COVID-19 in order to expand his vaccination programs.

COVID-19 was developed as a military weapon (by China, the United States, or some other country).

The number of deaths from COVID-19 has been exaggerated as a way to restrict liberties in the United States.

A fifth statement referenced the explanation that is currently considered most plausible by much of the scientific community [39]:

SARS-Cov-2, the virus that causes COVID-19, likely originated in animals [like bats] and then spread to humans.

Finally, 2 additional misinformed statements about face masks were added for this study [6,40,41]:

Wearing a face mask for COVID-19 prevention can cause oxygen deficiency or carbon dioxide intoxication.

Face masks are probably not helpful in reducing COVID-19 spread in a community.

Statistical and logical classification of participants into latent profiles based on the believability of misinformation was demonstrated in our prior research [7]. However, the current study occurred 8 months later than the original study and included new statements about face masks. Thus, profiles were computed based on the data from this study without prespecifying the existence of any classes (see the Statistical Analysis section). Then, all participants were assigned a numeric variable corresponding with their latent profile membership.

#### Aim Three

Aim Three targeted the intervention’s effect on participants’ behavioral intentions to engage in the COVID-19 preventive behaviors recommended by the CDC at the time of study administration [8,42]. Questions were based on structured measurement of intentions using the Theory of Planned Behavior [43], with response options ranging from 1 (unlikely) to 7 (likely). We prespecified 6 intentions in the protocol:

Wash your hands often (or use a hand sanitizer that contains at least 60% alcohol).

Avoid close contact (stay at least 6 feet from other people).

Cover your mouth and nose with a mask when around others.

Cover coughs and sneezes.

Clean and disinfect frequently touched surfaces daily.

Monitor your health daily.

Intention to get vaccinated was not prespecified in the protocol but was added as the seventh behavioral intention prior to administration in response to availability of vaccination for some US residents.

As planned, overall preventive behavioral intentions were assessed using exploratory factor analysis (see the Statistical Analysis section) to determine the number of factors present and then by computing mean scores for each factor to serve as outcomes. Intention to get vaccinated was analyzed as an isolated outcome of interest in a separate study [44] but was also included as a preventive behavior in this study’s factor analysis.

Because they already had received at least one shot of the vaccine, 49 participants were not asked to respond to the question about intention to get vaccinated for COVID-19; data for those individuals were imputed as a 7 (likely). Sensitivity analyses were performed without imputing data for those 49 participants, which led to similar results and conclusions. Therefore, imputed results were used in analyses throughout the manuscript.

#### Covariates

Additional measures were added as covariates for analysis, as prespecified, including political orientation and religious commitment [7,45]; race, gender, age, and education level; whether the participant had been diagnosed with (or believed they had) COVID-19 [46]; perceived severity of contracting COVID-19 and perceived ability to avoid contracting COVID-19 [47]; and normative belief about friends’ and family’s avoidance of crowded areas [48].

Due to evolving circumstances in the United States during this study, a question about COVID-19 vaccination status was added after the protocol was published. It read, “Vaccines to prevent COVID-19 have been approved by the Food and Drug Administration for use in the United States. The vaccines will be available to different people at different times. Did you already get a COVID-19 vaccine (at least one shot)?”

### Statistical Analysis

We planned to recruit 1000 participants, which would allow detection of small differences (Cohen d=0.18) with 80% power and would be sufficient for both types of planned analysis, linear mixed models (LMM) and path analyses [8].

#### Aim One

The primary outcome for Aim One, the effect of the infographic intervention on trust in science, was analyzed using an LMM controlling for all covariates (see the Outcomes section) with a random intercept for the individual participant. The interaction between study condition (intervention/control) and time (pre/postintervention) was estimated using contrasts to obtain the difference-in-difference using Kenward-Roger degrees of freedom approximation.

#### Aim Two

For the first component of this aim, we examined believability profiles for narrative statements about COVID-19 using latent profile analysis. To select the number of classes, we reviewed the Akaike information criterion (AIC), Bayesian information criterion (BIC) and adjusted BIC, class size, entropy, and results from the Vuong-Lo-Mendell-Rubin likelihood ratio test (LMR) to examine improvements in model fit for *k* versus *k-1* classes.

Next, we assigned a “profile” value to each participant based on the profile to which they most closely belonged. That variable was used as an outcome in the prespecified path analysis for this aim, which investigated adjusted odds of being a member of a less-scientific profile by examining the direct effect of the intervention and the indirect effect of the intervention mediated by trust in science, controlling for all other covariates. Finally, we presented results in parallel, treating profile as a multinomial variable (single model) and treating it as a dummy variable (one model per identified profile).

To elucidate other potentially interesting connections between the study variables, we conducted an exploratory, unplanned multivariate logistic regression analysis using profile membership as the outcome variable. All other variables served as dependent predictors except pre-intervention trust in science and having a professional diagnosis of COVID-19, which were highly associated with postintervention trust and believing one had been infected by COVID-19, respectively.

#### Aim Three

To determine the format of the outcome variable for this aim, we first conducted exploratory factor analysis (maximum likelihood with varimax rotation) to decide whether it was appropriate to treat the behavioral intentions regarding preventive behaviors as a monotonic scale [8]. Identification of a solution was based on assessment of eigenvalues, parallel analysis, factor loadings, and 2-dimensional spatial inspection. The computed scale variable(s) were used as outcomes in the prespecified path analyses for this aim, which computed the direct effect of the intervention on intentions to perform preventive behaviors and the indirect effect of the intervention thereon mediated by misinformation belief profile, controlling for all other covariates.

We computed exploratory path analyses to assess the influence of trust in science on preventive behaviors, with a mediation pathway through believability profile membership, with other variables serving as covariates. These analyses were for informative purposes only and were not used to generate any causal inferences.

### Role of the Funding Source

The funders of the study had no role in data collection, analysis, interpretation, or writing of the report. As reported in the protocol, grant reviewers made suggestions to improve study rigor that were incorporated prior to study initiation. Grant reviews were published alongside the protocol [8].

## Results

### Sample Characteristics

**Figure 3:**
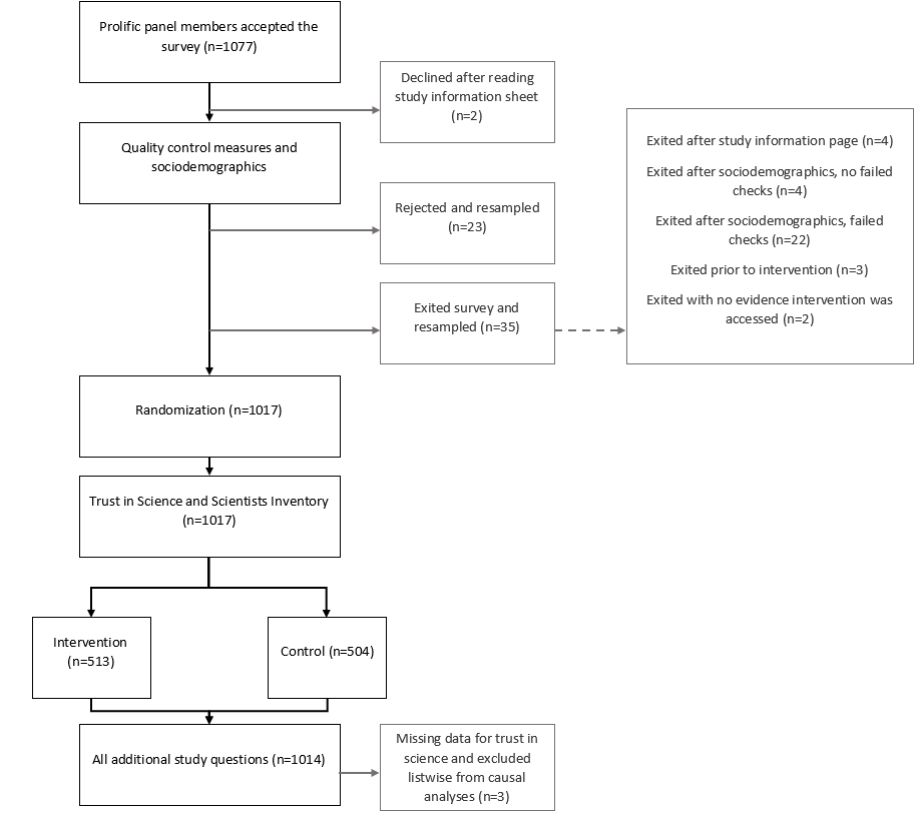
CONSORT flow diagram.

A representative panel of 1000 paid US respondents by gender, age, and race/ethnicity was solicited from Prolific on January 22, 2021 [35]. In total, 1077 Prolific panel members accepted the survey on the Prolific platform and accessed the Qualtrics study platform through January 24, 2021. The additional 77 cases included those who declined to participate after reading the study information sheet (n=2); who were rejected for failing a quality check (n=23); who exited the study (eg, closed their internet browser) prior to the intervention, most often immediately following a failed quality check (n=35); and who successfully completed the study but for unknown reasons did not request payment from Prolific (n=17). The latter 17 cases were retained for analysis in the arm to which they were randomly assigned, but random assignment beyond 1000 participants did not adhere to a 1:1 allocation ratio. The 3 cases who did not provide complete data for trust in science were excluded listwise from analyses except the latent profile computation for Aim 2. Thus, the final sample included 511 individuals randomized to the intervention arm and 503 individuals randomized to the control arm (Figure 3; Table 1).

**Table 1:** Sample characteristics by study arm.

Variable		Intervention (n=511)	Control (n=503)
**Gender, n (%)**			
	Male	251 (49.1)	238 (47.3)
	Female	254 (49.7)	261 (51.9)
	Nonbinary	3 (0.6)	3 (0.6)
	Transgender	3 (0.6)	1 (0.2)
**Race, n (%)**			
	White	388 (75.9)	394 (78.3)
	Black or African American	74 (14.5)	58 (11.5)
	American Indian or Alaska Native	3 (0.6)	2 (0.4)
	Asian	35 (6.8)	37 (7.4)
	Native Hawaiian or Pacific Islander	0 (0.0)	1 (0.2)
	Other	11 (2.2)	11 (2.2)
Hispanic or Latino/^a^ (Yes), n (%)		28 (5.5)	35 (7.0)
**COVID-19 diagnosis from a professional, n (%)**			
	Yes	15 (2.9)	27 (5.4)
	No/unsure	496 (97.1)	476 (94.5)
Religious commitment (1=low to 10=high), mean (SD)		4.15 (3.45)	4.06 (3.35)
Political orientation (1=liberal to 10=conservative), mean (SD)		4.27 (2.78)	4.15 (2.71)
Vaccination intention^a^ (1=unlikely to 7=likely), mean (SD)		5.48 (2.14)	5.50 (2.10)
Seriousness of contracting COVID-19 (1=not at all serious to 10=very serious), mean (SD)		6.60 (2.72)	6.41 (2.61)
Confidence avoiding COVID-19 (1=not very confident to 5=very confident), mean (SD)		3.25 (0.98)	3.27 (0.97)
Family/friends COVID-19 avoidance (1=strongly disagree to 7=strongly agree), mean (SD)		5.56 (1.51)	5.72 (1.40)
Age (years), mean (SD)		45.50 (16.61)	45.28 (16.19)

^a^Data do not include imputed values of “7” for vaccinated individuals.

### Aim One (Primary Outcome)

**Figure 4:**
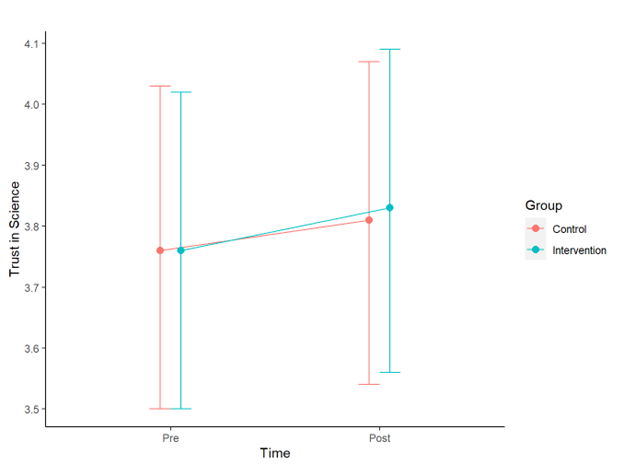
Trust in science scores and 95% CIs.

We hypothesized that exposure to the infographic intervention would have a moderate, positive effect on trust in science. This hypothesis was partly upheld. Our difference-in-difference analysis suggested that, controlling for all covariates, viewing the intervention infographic had a small, positive effect (0.03, SE 0.01, t_1000_=2.16, *P*=.031) on trust in science (Table 2; Figure 4). Additional details from the model as well as parceled analytic code are available in Multimedia Appendix 1.

**Table 2:** Contrast estimates for aim one.

Contrast	Estimate	SE	df	*t*	*P*
Control pre vs intervention pre	0.00	0.03	1048.17	0.10	>.999
Control pre vs control post	–0.04	0.01	1000.00	–5.11	<.001
Intervention pre vs intervention post	–0.07	0.01	1000.00	–8.23	<.001
Control post vs intervention post	–0.02	0.03	1048.17	–0.69	.90
Difference-in-difference (control pre-post) vs (intervention pre-post)	0.03	0.01	1000.00	2.16	.031

### Aim Two (Primary Outcome)

#### Computation of the Outcome Variable (Narrative Believability Profiles)

**Figure 5:**
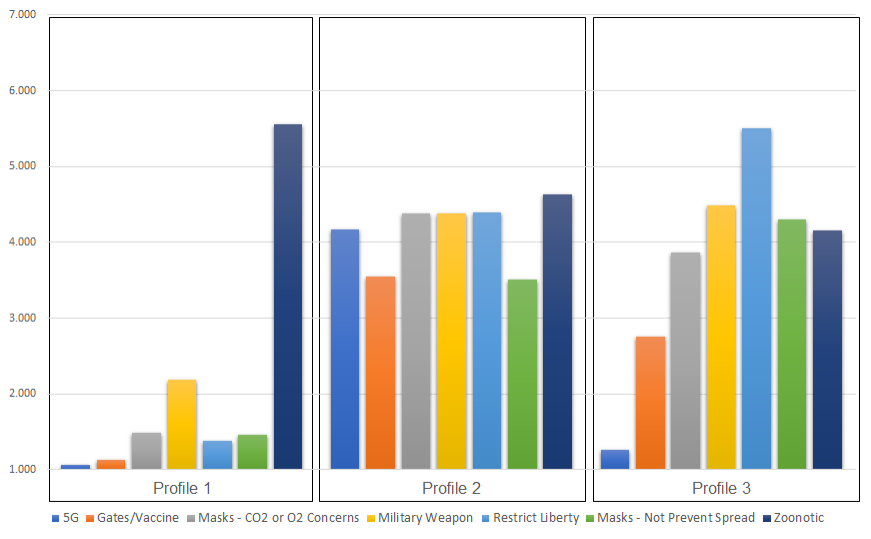
Believability of narrative statements by latent profile. Believability scores range from 1 (Extremely unbelievable) to 7 (Extremely believable).

Based on fit statistics (see Multimedia Appendix 1 for analytic code), we selected a 3-class model for use in this study. The primary metrics used to make this decision were the LMR test, entropy, and correspondence with extant data and prior studies. Table 3 and Figure 5 demonstrate the believability of narrative statements across each of the 3 profiles.

**Table 3:** Standardized means for latent profiles of narrative believability^a^ for aim two.

Statement	Profile One (828/1017, 81.42%)	Profile Two (42/1017, 4.13%)	Profile Three (147/1017, 14.45%)
5G	1.06	4.17	1.26
Gates/vaccine	1.14	3.54	2.75
Masks—CO2 or O2 concerns	1.49	4.38	3.86
Military weapon	2.19	4.38	4.48
Restrict liberty	1.39	4.39	5.51
Masks—not prevent spread	1.47	3.51	4.31
Zoonotic	5.55	4.63	4.15

^a^Believability scores ranged from 1 (Extremely unbelievable) to 7 (Extremely believable).

Profile One (828/1017, 81.42%), the largest class, was most likely to believe the zoonotic narrative (mean 5.55) and found most other narratives to be extremely unbelievable (mean <1.50), with the exception of the military weapon narrative (mean 2.19).

Profile Two (42/1017, 4.13%) was the smallest class and considered all the narratives to be moderately plausible, within a narrow band of believability scores (mean >3.50 and *<*4.65).

Profile Three (147/1017, 14.45%) reported differential believability across narratives. Members reported that the 5G theory (mean 1.26) and Bill Gates/vaccine narrative (mean 2.75) were extremely or mostly unbelievable. The misinformed idea that face masks can cause carbon dioxide intoxication or oxygen deficiency was perceived to be somewhat more believable (mean 3.86), as were the scientifically implausible statements that masks are not helpful in reducing COVID-19 spread (mean 4.31) or that COVID-19 was developed as a military weapon (mean 4.48). Believability of the zoonotic narrative also fell within this range (mean 4.15). For this profile, the most believable narrative was that the number of deaths from COVID-19 was exaggerated as a way to restrict liberties in the United States (mean 5.51).

#### Impact of the Intervention on Profile Membership

**Figure 6:**
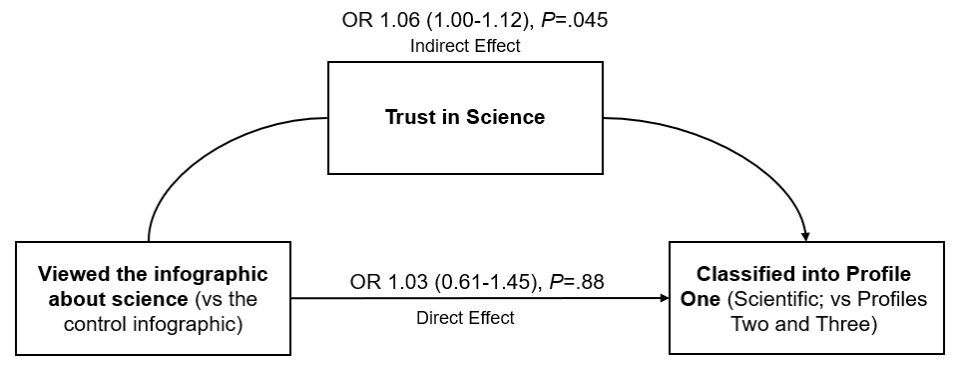
Influence of the intervention on the likelihood of being classified in Profile One, adjusted for age, gender, race, vaccination status, political orientation, perceived severity, perceived susceptibility, family behavior, prior diagnosis, prior infection, and pre-intervention trust. OR: odds ratio.

We hypothesized that exposure to the intervention would have a small, negative effect on the likelihood of belonging to a profile that believed misinformed or implausible narratives and that it would be partially mediated by trust in science. This hypothesis was partly upheld, as there was no evidence of a direct effect, but some evidence of a mediated effect (Table 4; Figure 6).

**Table 4:** Path analysis of the effects of the intervention on the believability profile.

Dependent variables		Odds ratio	SE	Lower CI	Upper CI	z	*P* value	AIC^a^
**Multinomial analysis: Profile Two^b^**								416.88
	Direct effect	0.96	0.35	0.28	1.64	–0.10	.92	
	Indirect effect	0.92	0.04	0.84	1.00	–1.95	.051	
	Total effect	0.89	0.32	0.25	1.52	–0.34	.74	
**Multinomial analysis: Profile Three^b^**								
	Direct effect	0.98	0.22	0.54	1.41	–0.11	.91	
	Indirect effect	0.95	0.02	0.90	1.00	–1.82	.07	
	Total effect	0.93	0.21	0.52	1.35	–0.32	.75	
**Binomial analysis: Profile One^c^**								
	Direct effect	1.03	0.21	0.61	1.45	0.16	.88	214.38
	Indirect effect	1.06	0.03	1.00	1.12	2.01	.045	
	Total effect	1.10	0.23	0.65	1.54	0.44	.66	
**Binomial analysis: Profile Two^c^**								
	Direct effect	1.00	0.35	0.31	1.70	0.01	.99	–147.42
	Indirect effect	0.93	0.04	0.86	1.00	–1.84	.07	
	Total effect	0.94	0.33	0.28	1.59	–0.19	.85	
**Binomial analysis: Profile Three^c^**								
	Direct effect	1.00	0.22	0.56	1.43	0.00	1.00	146.46
	Indirect effect	0.97	0.02	0.93	1.01	–1.56	.12	
	Total effect	0.97	0.22	0.55	1.39	–0.15	.88	

^a^AIC: Akaike information criterion.

^b^Reference is Profile One.

^c^Each profile is a dummy variable.

In the multinomial analysis, controlling for all covariates, the direct effect of viewing the intervention on belonging to Profile Two (versus Profile One) was nonsignificant (adjusted odds ratio [AOR] 0.96, SE 0.35, 95% CI 0.28-1.64, *z*=–0.10, *P*=.92), but there was some evidence of a marginal indirect effect mediated by trust in science (AOR 0.92, SE 0.04, 95% CI 0.84-1.00, *z*=–1.95, *P*=.051). Results for Profile 3 (versus Profile One) were similar, with a nonsignificant direct effect (AOR 0.98, SE 0.22, 95% CI 0.54-1.41, *z*=–0.11, *P*=.91) and limited evidence of a marginal indirect effect mediated by trust in science (AOR 0.95, SE 0.02, 95% CI 0.90-1.00, *z*=–1.82, *P*=.07).

To support disambiguation of the indirect effect, we also conducted binomial path analyses using each profile as a dummy variable. The direct effect of viewing the intervention on belonging to Profile One was nonsignificant (AOR 1.03, SE 0.21, 95% CI 0.61-1.45, *z*=0.16, *P*=.88), but there was evidence of a small indirect effect mediated by trust in science (AOR 1.06, SE 0.03, 95% CI 1.00-1.12, *z*=2.01, *P*=.045). Full output from the model and analytic code are available in Multimedia Appendix 1.

### Aim Three (Primary Outcome)

#### Computation of the Outcome Variable (COVID-19 Preventive Behaviors)

Exploratory factor analysis did not clearly indicate whether the 7 preventive behaviors formed a monotonic or 2-factor scale. Discrimination based on eigenvalues favored a 2-factor solution, which cumulatively explained 46% of the variance (χ^2^_8_=124.1, *P*<.001), while parallel analysis favored a 1-factor solution, which explained 37% of the variance (χ^2^_14_=357.1, *P*<.001). Conceptually, both approaches were logical.

In the 2-factor solution, handwashing, cleaning and disinfecting surfaces daily, and monitoring one’s health daily cleanly loaded on factor 1, while avoiding close contact, covering one’s mouth and nose with a mask when around others, and getting vaccinated for COVID-19 loaded on factor 2, with covering coughs and sneezes loading weakly on both factors, but more strongly (0.41) on factor 1. The 95% CIs for the Cronbach alpha were 0.68-0.73 for factor 1 and 0.64-0.71 for factor 2. In the 1-factor solution, variable loadings ranged from 0.48 to 0.71, and the 95% CIs for the Cronbach alpha was 0.74-0.79.

As prespecified [8], the factor analysis guided further analyses for this aim. Given the conceptual complexity, we opted to complete separate analyses for both 1-factor and 2-factor preventive behavior solutions and to interpret them in tandem.

#### Impact of the Intervention on Behavioral Intentions

**Figure 7:**
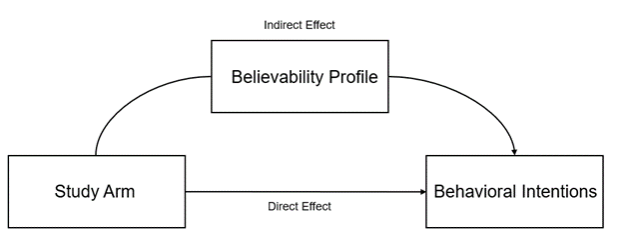
Hypothesized causal pathway of the intervention (not supported), adjusted for age, gender, race, vaccination status, political orientation, perceived severity, perceived susceptibility, family behavior, prior diagnosis, prior infection, and pre-intervention trust.

We hypothesized that exposure to the intervention would have a small, positive effect on behavioral intentions that would be partially mediated by believability profile membership (Figure 7). Controlling for all covariates, we found no evidence that the intervention affected behavioral intentions to engage in COVID-19 preventive behavior using either a 1-factor or 2-factor outcome (Tables 5, 6, and 7).

**Table 5:** Path analysis of effects of the intervention on preventive behaviors (1-factor solution).

Mediators		Coefficient	SE	Lower CI	Upper CI	z	*P* value	AIC^a^
**Multinomial analysis: Profile Two^b^**								
	Direct effect	–0.04	0.04	–0.12	0.05	–0.85	.39	3018.13
	Indirect effect	0.05	0.22	–0.38	0.49	0.23	.82	
	Total effect	0.01	0.23	–0.43	0.46	0.06	.95	
**Multinomial analysis: Profile Three^b^**								
	Direct effect	–0.04	0.04	–0.12	0.05	–0.85	.39	
	Indirect effect	0.04	0.15	–0.26	0.33	0.24	.82	
	Total effect	0.00	0.16	–0.30	0.30	0.00	1.00	
**Binomial analysis: Profile One^c^**								
	Direct effect	–0.04	0.04	–0.12	0.05	–0.86	.39	2814.37
	Indirect effect	0.05	0.13	–0.22	0.31	0.35	.73	
	Total effect	0.01	0.14	–0.27	0.29	0.07	.94	
**Binomial analysis: Profile Two^c^**								
	Direct effect	–0.03	0.05	–0.12	0.06	–0.70	.48	2528.63
	Indirect effect	0.01	0.15	–0.28	0.31	0.10	.92	
	Total effect	–0.02	0.16	–0.32	0.29	–0.11	.91	
**Binomial analysis: Profile Three^c^**								
	Direct effect	–0.03	0.04	–0.12	0.05	–0.76	.45	2763.44
	Indirect effect	0.01	0.13	–0.24	0.27	0.08	.93	
	Total effect	–0.02	0.14	–0.29	0.25	–0.17	.87	

^a^AIC: Akaike information criterion.

^b^Reference is Profile One.

^c^Each profile is a dummy variable.

**Table 6:** Multinomial path analysis of the effects of the intervention on preventive behaviors (2-factor solution), in which the reference is Profile One.

Mediator		Coefficient	SE	Lower CI	Upper CI	z	*P* value	AIC^a^
**Factor 1^b^as the dependent variable: Profile Two**								3376.55
	Direct effect	–0.03	0.05	–0.13	0.07	–0.62	.53	
	Indirect effect	0.03	0.13	–0.22	0.28	0.23	.82	
	Total effect	0.00	0.14	–0.27	0.26	–0.02	.98	
**Factor 1^b^ as the dependent variable: Profile Three**								
	Direct effect	–0.03	0.05	–0.13	0.07	–0.62	.53	
	Indirect effect	0.02	0.07	–0.12	0.15	0.24	.81	
	Total effect	–0.02	0.09	–0.18	0.15	–0.18	.86	
**Factor 2^c^ as the dependent variable: Profile Two**								3440.75
	Direct effect	–0.04	0.05	–0.15	0.06	–0.81	.42	
	Indirect effect	0.08	0.35	–0.61	0.77	0.23	.82	
	Total effect	0.04	0.35	–0.66	0.73	0.11	.92	
**Factor 2^c^ as the dependent variable: Profile Three**								
	Direct effect	–0.04	0.05	–0.15	0.06	–0.81	.42	
	Indirect effect	0.06	0.26	–0.44	0.57	0.24	.81	
	Total effect	0.02	0.26	–0.49	0.53	0.07	.94	

^a^AIC: Akaike information criterion.

^b^Preventive behaviors 1, 4, 5, and 6.

^c^Preventive behaviors 2, 3, and 7.

**Table 7:** Binomial path analysis of the effects of the intervention on preventive behaviors (2-factor solution), in which each profile is a dummy variable.

Mediator			Coefficient		SE		Lower CI		Upper CI		z		*P* value		AIC^a^
**Factor 1^b^ as the dependent variable: Profile One**															
	Direct effect	–0.03		0.05		–0.13		0.07		–0.62		.53		3172.83	
	Indirect effect	0.02		0.06		–0.10		0.15		0.35		.73			
	Total effect	–0.01		0.08		–0.17		0.15		–0.12		.91			
**Factor 1^b^ as the dependent variable: Profile Two**															
	Direct effect	–0.03		0.05		–0.13		0.07		–0.57		.57		2820.14	
	Indirect effect	0.01		0.09		–0.17		0.19		0.09		.92			
	Total effect	–0.02		0.11		–0.23		0.19		–0.20		.84			
**Factor 1^b^ as the dependent variable: Profile Three**															
	Direct effect	–0.03		0.05		–0.13		0.07		–0.58		.56		3100.05	
	Indirect effect	0.00		0.06		–0.11		0.12		0.08		.93			
	Total effect	–0.03		0.08		–0.18		0.13		–0.33		.74			
**Factor 2^c^ as the dependent variable: Profile One**															
	Direct effect	–0.04		0.05		–0.15		0.06		–0.81		.42		3237.87	
	Indirect effect	0.08		0.23		–0.36		0.52		0.35		.73			
	Total effect	0.04		0.23		–0.42		0.49		0.15		.88			
**Factor 2^c^ as the dependent variable: Profile Two**															
	Direct effect	–0.03		0.06		–0.15		0.08		–0.59		.56		3020.59	
	Indirect effect	0.02		0.22		–0.42		0.46		0.10		.92			
	Total effect	–0.01		0.23		–0.47		0.44		–0.05		.96			
**Factor 2^c^ as the dependent variable: Profile Three**															
	Direct effect	–0.04		0.05		–0.15		0.07		–0.69		.49		3203.54	
	Indirect effect	0.02		0.23		–0.42		0.46		0.08		.93			
	Total effect	–0.02		0.23		–0.48		0.44		–0.08		.94			

^a^AIC: Akaike information criterion.

^b^Preventive behaviors 1, 4, 5, and 6.

^c^Preventive behaviors 2, 3, and 7.

### Secondary Outcomes

#### Exploratory Multivariate Logistic Regression on Profile Membership

Independent of the path analysis, we investigated what factors were associated with classification in each of the 3 belief profiles. Each 1-point movement toward political conservatism on a 10-point scale was associated with 1.39 adjusted odds of belonging to Profile Three versus Profile One (χ^2^_1_=40.52, 95% CI 1.25-1.53, *P*<.001), and each 1-point increase in perceived severity of COVID-19 was associated with 0.82 adjusted odds of belonging to Profile Three versus Profile One (χ*^2^*_1_=19.04, 95% CI 0.75-0.90, *P*<.001). Each additional year of age was associated with a slight decrease in adjusted odds (0.972) of belonging to Profile Two versus Profile One (χ^2^_1_=4.65, 95% CI 0.95-1.00, *P*=.031).

Finally, each 1-point increase in trust in science was associated with substantially lower adjusted odds of belonging to Profile Three (0.21) or Profile Two (0.14) compared with Profile One (Profile Three: χ^2^_1_=55.57, 95% CI 0.14-0.31, *P*<.001; Profile Two: χ^2^_1_=39.20, 95% CI 0.08-0.26, *P*<.001). See Multimedia Appendix 1 and Multimedia Appendix 2 for all outputs and code.

#### Exploratory Path Analysis of the Association Between Trust in Science and Preventive Behavioral Intentions

We computed exploratory path analyses to assess the influence of trust in science on preventive behaviors, with a mediation pathway through believability profile membership. In the 1-factor preventive behavior model treating believability Profile One as a dummy variable, there was a significant direct effect (0.46, SE 0.11, 95% CI 0.24-0.69, *z*=4.05, *P*<.001) of trust in science on preventive behavior, as well as a significant indirect effect mediated by believability profile (1.28, SE 0.36, 95% CI 0.57-1.99, *z*=3.53, *P*<.001). Similar outcomes were observed for the 2-factor model (see Multimedia Appendix 1 and Multimedia Appendix 2). These specific findings were correlational, not causal.

## Discussion

This study provides preliminary evidence and a proof-of-concept for using infographics that truthfully address underlying reasons why a person might not trust science or scientists to (1) improve trust in science and (2) provide inoculation against COVID-19 misinformation. However, observed effects were small, as expected for a short, passive, and inexpensive intervention. Much remains to be learned in this area of research. Here, we discuss the main findings separately by study aim, provide additional interpretation of exploratory analyses, and make recommendations for future work.

### Aim One

#### Principal Finding

This study found that viewing an infographic designed to truthfully address underlying reasons why a person might not trust science or scientists [37] once, for a minimum of 60 seconds, caused a small aggregate increase in participants’ overall trust in science.

#### Interpretation

The scale used in this study measured trust in science as a composite from 21 questions to yield a score from 1 to 5. We posit that the small difference-in-difference improvement estimate (+0.03) is meaningful due to the simplicity of the intervention and the ease with which such an intervention could be deployed to large numbers of people. Especially given recent research indicating that aggregate social trust in science may affect variables like vaccine confidence beyond individual-level trust [49], we believe this finding merits replication research. At the same time, though trust in science is a worthy concept to study in and of itself, we were particularly interested in the degree to which changing trust might affect misinformation or behavioral intentions. This aim simply established that it is plausible that a brief, single exposure to an infographic can improve trust in science.

### Aim Two

#### Principal Finding

We found some evidence that viewing an infographic designed to truthfully address underlying reasons why a person might not trust science or scientists [37] once, for a minimum of 60 seconds, may have had a very small indirect effect on belief in COVID-19 misinformation.

#### Interpretation

There is ongoing discussion among methodologists and metascientists as to how to interpret mediation effects in terms of causal attribution, especially when the direct and total effects are nonsignificant. In general, we can think of a direct effect as proposing that “*X* is regularly followed by *Y,*” while an indirect effect suggests “*Y* if and only if *A*” [50]. Here, *X* is “viewing the infographic about science,” and *Y* is “belonging to the scientific latent belief profile,” while *A* is “an increase in trust in science and scientists.” We did not find any evidence of a direct effect or total effect—that is, viewing our intervention infographic was not regularly followed by increased likelihood of belonging to the scientific profile, nor was it sufficient for establishing increased likelihood of belonging to that profile. However, the mediation effect (in this case, OR 1.06) suggests that viewing the intervention resulted in increased likelihood of belonging to the scientific profile *if and only if* viewing the intervention also increased trust in science to a sufficient degree. In conjunction with the main finding from Aim One, this supports the plausibility of the intervention functioning in this manner.

Because this study used a randomized, controlled experimental design and included numerous covariates, endogeneity bias was not a substantive concern in interpretation [51,52]. Further, the sample was drawn to be nationally representative of the US population by age, sex, race, and ethnicity [35], attenuating but not eliminating concerns about generalizability [53]. Given the complexity of the topic, further investigation of this relationship as well as experimental replication are both needed before drawing any sort of definitive conclusion.

That noted, we encourage such research to be undertaken with some urgency. This work has meaningful, practical application if the findings hold true. While fact checking can reduce belief in misinformation, it is not likely feasible to respond to the amount and variability that is produced, even for a single topic like COVID-19 [27]. Further, the type and nature of misinformation can rapidly shift in unexpected ways. For example, during revision of this manuscript, the Mississippi State Department of Health issued a warning that 70% of recent calls to their Poison Control Center were related to ingesting livestock or animal formulations of ivermectin [54]. The comparative advantage of the type of trust-mediated prophylaxis proposed and tested here (versus debunking or fact checking) is that it does not require addressing each new misinformed claim. In that sense, such interventions affecting believability of misinformation might work well alongside interventions to reduce the likelihood of sharing it [32,33].

### Aim 3

#### Principal Finding

Viewing an infographic designed to truthfully address underlying reasons why a person might not trust science or scientists [37] once, for a minimum of 60 seconds, did not significantly affect behavioral intentions to engage in COVID-19 preventive behaviors.

#### Interpretation

We hypothesize, but cannot be certain, that this null finding emerged because this specific infographic addressed a component of rational epistemic trust (eg, why it makes sense to trust scientific findings even when they change over time) [16] but did not address recommendation trust (eg, did not offer any reassurance that scientists make recommendations that are in the best interests of others) [19]. Our exploratory analyses did indicate a significant, strong association between trust in science and COVID-19 preventive behavioral intentions, indicating the plausibility that a strategic effect targeting that recommendation trust might affect such intentions. It is also possible, of course, that behavioral intentions are sufficiently complex and difficult to change that a single infographic viewing, regardless of content, would not “move the needle” [55].

### Exploratory Findings

This study also identified potentially valuable information about how beliefs about COVID-19 may cluster. In May 2020, we identified a single latent profile that endorsed the zoonotic narrative and generally found other narratives unbelievable and 3 profiles that believed misinformed narratives to varying degrees but also believed the zoonotic narrative [7]. The present study was conducted in a different information ecosystem (around 8 months later) and included 2 additional narratives about face masks.

Two major findings about profiles were consistent between our studies: There was a single profile endorsing the zoonotic narrative and generally disbelieving other narratives, and no narrative profile rejected the zoonotic explanation. However, there were only 2 nonscientific profiles in this study rather than 3, and interpretation of their meaning was clearer than in the original study. The smallest profile (Profile Two) found all narratives to be at least somewhat believable. In contrast, Profile Three was comparatively less likely to endorse narratives that were subjectively less political in the United States (eg, 5G, Gates/vaccine) and more likely to believe other narratives (eg, restrict liberty, masks don’t prevent spread). It is unclear whether this difference was due to the addition of the face mask narratives, a change in the information ecosystem, the use of a nationally representative sample, or a different reason altogether.

Notably, in our unplanned regression analysis, trust in science remained the most substantive predictor of profile membership, as in May 2020. However, unlike our previous study, in which political orientation was not associated with profile membership, here we found that conservative political orientation was associated with classification in Profile Three versus Profile One, but not with classification in Profile Two. Along with the profile analysis itself, this suggests the possibility of 2 “typologies” of misinformation belief, 1 that is apolitical and may believe even scientifically impossible narratives (eg, finding all narratives to be plausible) and 1 that is associated with political orientation and that believes misinformation somewhat selectively, applying an alternate decision heuristic in determining what is plausible.

### Limitations and Future Directions

This study investigated multiple outcomes and so there was some increased risk of Type 1 error. For this reason, we interpreted the outcomes cautiously and recommend replication prior to any definitive determination about these findings. At the same time, the primary outcomes were prespecified and were assessed using a limited number of models. A limitation specific to the third aim is that behavioral intentions are not behaviors, so this study should not be interpreted to assess the effect of the intervention on actual behavior. In addition, we opted to limit the allowable content in the intervention. As we note in our pilot study [37], we very purposefully used messaging about science and scientists that we believed to be truthful. Our intention specifically was not to “manipulate” trust in science but rather to determine whether exposure to an easily digested, truthful accounting had a causal effect.

There were numerous decisions made in the course of developing the single image used as the intervention in this study, as well as the structure of the intervention. Given this proof-of-concept, there is much room to explore alternative approaches, including, but not limited to, investigating whether a brief video would be more efficacious than a static image, the art style or amount of wording matters, embedding the image as an ad in social media (eg, repeated natural exposures) over a period of time affects the results, and comparison to real negative messages about science would produce similar results to this study, which used an active placebo about dogs.

## Appendix

Multimedia Appendix 1 All analytic code and instructions for its use to replicate results and generate additional tables.

Multimedia Appendix 2 CSV datasets used with Appendix 1.

Multimedia Appendix 3 CONSORT-eHEALTH checklist (V 1.6.1).

## Abbreviations

AIC: Akaike information criterion
AOR: adjusted odds ratio
BIC: Bayesian information criterion
CDC: Centers for Disease Control and Prevention
LMM: linear mixed model
LMR: Vuong-Lo-Mendel-Rubin Likelihood Ratio Test
NPB: nonpharmaceutical preventive behaviors
OR: odds ratio
