# Supplementary material for: Intervening on Trust in Science to Reduce Belief in COVID-19 Misinformation and Increase COVID-19 Preventive Behavioral Intentions: Randomized Controlled Trial
Source: J Med Internet Res. 2021 Oct 14;23(10):e32425. doi: 10.2196/32425 (PMC8519341; doi:10.2196/32425)
Supplement: Multimedia Appendix 1 [file jmir_v23i10e32425_app1.docx]

**Appendix for “Effects of briefly viewing an infographic about science on trust in science, belief in COVID-19 misinformation, and COVID-19 preventive behavioral intentions: a two-arm, parallel group randomized controlled trial”**

**Information for Using this Appendix**

This document, along with the associated data files (see below), will allow generation of all charts and outputs used in the preparation of this manuscript. We have separated syntaxes by function and Aim. You must run the Data Cleaning Syntax first for some subsequent analyses to be run. The last syntax (in Mplus) was used to compute the latent profile information that is included in the ‘misinformation_LPA.csv’ dataset using the raw data as input.

Software required:

R (R version 4.1.0 and RStudio version 1.4.1717)
Stata (Stata version 16)

SAS (SAS version 9.4)

Mplus (Mplus version 7.3)

Raw data:

COVID Misinformation final.csv [direct download from Qualtrics]

misinformation_LPA.csv [dataset with latent profile class information added]

classprob.csv [same as above, but used for analysis on a different PC]

**Data Cleaning Syntax (R)**

#-#-# Load libraries

library(dplyr)

#-#-# Create function for not include

`%notin%` <- Negate(`%in%`)

#-#-# Set working directory

setwd("C:/ Insert Path to File Here /COVID misinformation")

#-#-# Read raw data

final.data <- read.csv("Data/Raw/COVID Misinformation final.csv")

LPA.data <- read.csv("Data/Raw/misinformation_LPA.csv")

#-#-# Data cleaning for survey data

final.data.clean <- final.data %>%

mutate(Rejected = RejectAttention1 + RejectHonesty + RejectVPN + RejectAttention2) %>%

filter(Rejected == 0) %>%

filter(SIS == 1) %>%

filter(Timer1_Page_Submit>0 | Timer2_Page_Submit>0) %>%

# Reverse scores for selected questions

mutate(Trust1_1_1 = 6-Trust1_1_1, Trust1_1_2 = 6-Trust1_1_2, Trust1_1_4 = 6-Trust1_1_4,

Trust1_1_5 = 6-Trust1_1_5, Trust1_1_7 = 6-Trust1_1_7, Trust1_1_9 = 6-Trust1_1_9,

Trust1_2_3 = 6-Trust1_2_3, Trust1_2_7 = 6-Trust1_2_7, Trust1_2_8 = 6-Trust1_2_8,

Trust1_2_9 = 6-Trust1_2_9, Trust1_2_10 = 6-Trust1_2_10, Trust1_2_11 = 6-Trust1_2_11,

Trust2_1_1 = 6-Trust2_1_1, Trust2_1_2 = 6-Trust2_1_2, Trust2_1_3 = 6-Trust2_1_3,

Trust2_1_4 = 6-Trust2_1_4, Trust2_1_6 = 6-Trust2_1_6, Trust2_1_8 = 6-Trust2_1_8,

Trust2_2_3 = 6-Trust2_2_3, Trust2_2_7 = 6-Trust2_2_7, Trust2_2_8 = 6-Trust2_2_8,

Trust2_2_9 = 6-Trust2_2_9, Trust2_2_10 = 6-Trust2_2_10, Trust2_2_11 = 6-Trust2_2_11) %>%

select(-Trust1_1_3) %>% #Trust 3, from PRE is an attention 3, so it is not part of the scale Trust1_1_3.

filter(RandomID %notin% c(44142400, 86337727, 16373208)) %>%

rowwise() %>%

# Calculate means for trust in science

mutate(Trust1 = sum(c_across(Trust1_1_1:Trust1_2_11))/21,

Trust2 = sum(c_across(Trust2_1_1:Trust2_2_11))/21) %>%

ungroup()

#-#-# Rename variables for LPA dataset

LPA.data.clean <- LPA.data %>%

rename(RandomID = ID, Profiles = C)

#-#-# Wide format data

# Merge wide data and calculate means for preventive behaviors

clean.data.wide <- merge(final.data.clean, LPA.data.clean, by = "RandomID", all.x = TRUE) %>%

mutate(NPB = rowMeans(select(., starts_with("NPB")), na.rm = TRUE),

NPB_f1 = rowMeans(select(., c("NPB_1", "NPB_4", "NPB_5", "NPB_6"))),

NPB_f2 = rowMeans(select(., c("NPB_2", "NPB_3", "NPB_7")), na.rm = TRUE)) %>%

#Data for those individuals who already had received at least one shot of the vaccine will be imputed as a 7 ("likely").

mutate(NPB_7_i = ifelse(is.na(NPB_7), 7, NPB_7)) %>%

mutate(NPB_i = rowMeans(select(., c("NPB_1", "NPB_2", "NPB_3", "NPB_4", "NPB_5", "NPB_6", "NPB_7_i"))),

NPB_f1_i = rowMeans(select(., c("NPB_1", "NPB_4", "NPB_5", "NPB_6"))),

NPB_f2_i = rowMeans(select(., c("NPB_2", "NPB_3", "NPB_7_i")))) %>%

select(c("RandomID", "arm", "Age", "Gender", "Race", "Ethnicity",

"Profiles",

"NPB_1", "NPB_2", "NPB_3", "NPB_4", "NPB_5", "NPB_6", "NPB_7", "NPB_7_i",

"NPB", "NPB_f1", "NPB_f2", "NPB_i", "NPB_f1_i", "NPB_f2_i",

"Vaccination", "Religious", "Political", "Severity", "SelfEfficacy", "FamBehav", "Diagnose", "Infect",

"Trust1", "Trust2"))

#-#-# Long format data

# Create one data set for pre data

clean.data.pre <- clean.data.wide[,1:30] %>%

rename(Trust = Trust1) %>%

mutate(Time = 0)

# Create one data set for post data

clean.data.post <- clean.data.wide[,c(1:29,31)] %>%

rename(Trust = Trust2) %>%

mutate(Time = 1)

# Merge pre and post to obtain long data set for linear mixed model

clean.data.long <- rbind(clean.data.pre, clean.data.post) %>%

arrange(RandomID, Time)

#-#-# Save clean data sets

write.csv(clean.data.wide, "Data/Cleaned/COVID Misinformation clean wide.csv", row.names = FALSE, na = "")

write.csv(clean.data.long, "Data/Cleaned/COVID Misinformation clean long.csv", row.names = FALSE, na = "")

**Data Analysis for Aim One and EFA for Aim Three (R)**

---

title: "#Misinformation"

author: "Xiwei Chen"

date: "`r format(Sys.time(), '%Y %B %d')`"

output:

html_document:

df_print: paged

code_folding: hide

---

_files:_\

COVID Misinformation clean wide.csv\

COVID Misinformation clean long.csv

```{r setup, include=FALSE}

knitr::opts_chunk$set(echo = TRUE)

# Load libraries

library(dplyr)

library(openxlsx)

library(ggplot2)

library(lme4); library(lmerTest); library(car); library(emmeans)

library(psych); library(GPArotation); library(nFactors)

# Read wide data and set variables as factors

data.wide <- read.csv("C:/ Insert Path to File Here /Data/Cleaned/COVID Misinformation clean wide.csv") %>%

mutate(arm = factor(arm, levels = c(0,1), labels = c("Control", "Intervention")),

Gender = as.factor(Gender),

Race = as.factor(Race),

Ethnicity = as.factor(Ethnicity),

Profiles = as.factor(Profiles),

Vaccination = as.factor(Vaccination),

Diagnose = as.factor(Diagnose),

Infect = as.factor(Infect))

# Read long data and set variables as factors

data.long <- read.csv("C:/ Insert Path to File Here/Data/Cleaned/COVID Misinformation clean long.csv") %>%

mutate(arm = factor(arm, levels = c(0,1), labels = c("Control", "Intervention")),

Time = factor(Time, levels = c(0,1), labels = c("Pre", "Post")),

Gender = as.factor(Gender),

Race = as.factor(Race),

Ethnicity = as.factor(Ethnicity),

Profiles = as.factor(Profiles),

Vaccination = as.factor(Vaccination),

Diagnose = as.factor(Diagnose),

Infect = as.factor(Infect))

```

# Aim 1

<style>

div.blue { background-color:#e6f0ff; border-radius: 5px; padding: 20px;}

</style>

<div class = "blue">

Linear mixed model (LMM) was used to examine the effect of the intervention on trust in science, controlling for specified covariates (Age, Gender, Race, Vaccination, Religious, Political, Severity, SelfEfficacy, FamBehav, Diagnose, Infect), with random effect of RandomID.

</div>

```{r}

fitlmm <- lmer(Trust ~ arm * Time + Age + Gender + Race +

Vaccination + Religious + Political + Severity + SelfEfficacy + FamBehav + Diagnose + Infect + (1|RandomID),

data = data.long,

contrasts=list(arm=contr.sum, Time=contr.sum, Gender=contr.sum, Race=contr.sum,

Vaccination=contr.sum, Diagnose=contr.sum, Infect=contr.sum))

```

#### Type 3 test

```{r}

type3 <- data.frame(Anova(fitlmm, type="III")) %>%

mutate(Variable = rownames(.),

Chisq = round(Chisq,2),

P.value = ifelse(Pr..Chisq.<0.001, "<0.001", round(Pr..Chisq.,3))) %>%

select(4,1,2,5)

type3

```

#### Coefficient for random effect

```{r}

random.coef <- data.frame(VarCorr(fitlmm)) %>%

mutate(vcov = round(vcov,2),

sdcor = round(sdcor,2)) %>%

select(-var2)

random.coef

```

#### Coefficient for fix effect

```{r}

fix.coef <- data.frame(summary(fitlmm)$coef) %>%

mutate(Effect = rownames(.),

Estimate = round(Estimate,2),

Std.Error = round(Std..Error,2),

df = round(df,0),

t.value = round(t.value,2),

P.value = ifelse(Pr...t..<0.001, "<0.001", round(Pr...t..,3))) %>%

select(6,1,7,3,4,8)

fix.coef

```

#### Estimate means and contrasts

```{r}

em <- emmeans(fitlmm, pairwise ~ arm*Time)

emmean <- data.frame(em$emmeans) %>%

mutate(emmean = round(emmean,2),

SE = round(SE,2),

lower.CL = round(lower.CL,2),

upper.CL = round(upper.CL,2))

emmean

contrast <- data.frame(em$contrasts) %>%

mutate(estimate = round(estimate,2),

SE = round(SE,2),

t.ratio = round(t.ratio,2),

P.value = ifelse(p.value<0.001, "<0.001", round(p.value,3))) %>%

select(-p.value)

contrast

```

#### Difference-in-Difference

```{r}

pairs(pairs(emmeans(fitlmm, ~ Time|arm)), by = NULL)

```

#### Plot

```{r}

plot <- emmean %>%

ggplot(aes(x = Time, group = arm, color = arm)) +

geom_errorbar(aes(ymin = lower.CL, ymax = upper.CL), position = position_dodge(0.2), width = 0.2) +

geom_line(aes(y = emmean, color = arm), position = position_dodge(0.2)) +

geom_point(aes(y = emmean, color = arm), position = position_dodge(0.2), size = 2.5) +

labs(y = "Trust in Science", color = "Arm") +

theme(panel.background = element_blank(), axis.line = element_line(colour = "black"))

plot

# tiff("C:/Users/Xino/Indiana University/O365-BCC - Documents/Projects/Agley, Jon/COVID misinformation/Output/Fig emmean.tif",

# units="in", width=6, height=4, res=500, compression = "lzw")

# plot

# dev.off()

```

# Aim 3 - Exploratory factor analysis (EFA)

<style>

div.blue { background-color:#e6f0ff; border-radius: 5px; padding: 20px;}

</style>

<div class = "blue">

Exploratory factor analysis (EFA) was performed to determine whether the intention-based items (NPB 1 to 7) to function as a monotonic scale that can be collapsed into a single variable.

</div>

## Using original item 7 (NPB_7)

```{r, include=FALSE}

# https://www.statmethods.net/advstats/factor.html

# https://www.promptcloud.com/blog/exploratory-factor-analysis-in-r/

NPB.data <- data.wide[,c("NPB_1", "NPB_2", "NPB_3", "NPB_4", "NPB_5", "NPB_6", "NPB_7")]

NPB.data.complete <- NPB.data[complete.cases(NPB.data),]

NPB.cor <- cor(NPB.data.complete)

```

### Determining the number of factors to extract

#### Eigenvalues

```{r}

ev <- eigen(NPB.cor)

ev$values

```

#### Plot

```{r}

ap <- parallel(subject=nrow(NPB.data.complete), var=ncol(NPB.data.complete), rep=100, cent=.05) #Parallel analysis

nS <- nScree(x=ev$values, aparallel=ap$eigen$qevpea)

plotnScree(nS)

```

<style>

div.blue { background-color:#e6f0ff; border-radius: 5px; padding: 20px;}

</style>

<div class = "blue">

Both eigenvalues & parallel analysis suggest that n factor = 2.

</div>

### EFA (Maximum likelihood factor analysis with varimax rotation)

#### 2 factors

```{r}

NPB.fa.2 <- factanal(NPB.data.complete, factors = 2, rotation = "varimax")

print(NPB.fa.2, digits=2, cutoff=.3, sort=FALSE)

plot(NPB.fa.2$loadings,type="n"); text(NPB.fa.2$loadings,labels=names(NPB.data.complete),cex=.7)

```

##### Cronbach's alpha

```{r}

alpha(NPB.data.complete[,c("NPB_1", "NPB_4", "NPB_5", "NPB_6")])

alpha(NPB.data.complete[,c("NPB_2", "NPB_3", "NPB_7")])

```

#### 1 factor

```{r}

NPB.fa.1 <- factanal(NPB.data.complete, factors = 1, rotation = "varimax")

print(NPB.fa.1, digits=2, sort=FALSE)

```

##### Cronbach's alpha

```{r}

alpha(NPB.data.complete[,c("NPB_1", "NPB_2", "NPB_3", "NPB_4", "NPB_5", "NPB_6", "NPB_7")])

```

## Using imputed item 7 (NPB_7_i)

```{r, include=FALSE}

# https://www.statmethods.net/advstats/factor.html

# https://www.promptcloud.com/blog/exploratory-factor-analysis-in-r/

NPB.data <- data.wide[,c("NPB_1", "NPB_2", "NPB_3", "NPB_4", "NPB_5", "NPB_6", "NPB_7_i")]

NPB.cor <- cor(NPB.data)

```

### Determining the number of factors to extract

#### Eigenvalues

```{r}

ev <- eigen(NPB.cor)

ev$values

```

#### Plot

```{r}

ap <- parallel(subject=nrow(NPB.data), var=ncol(NPB.data), rep=100, cent=.05) #Parallel analysis

nS <- nScree(x=ev$values, aparallel=ap$eigen$qevpea)

plotnScree(nS)

```

<style>

div.blue { background-color:#e6f0ff; border-radius: 5px; padding: 20px;}

</style>

<div class = "blue">

Eigenvalues suggests that n factor = 2, while parallel analysis suggests that n factor = 1.

</div>

### EFA (Maximum likelihood factor analysis with varimax rotation)

#### 2 factors

```{r}

NPB.fa.2 <- factanal(NPB.data, factors = 2, rotation = "varimax")

print(NPB.fa.2, digits=2, cutoff=.3, sort=FALSE)

plot(NPB.fa.2$loadings,type="n"); text(NPB.fa.2$loadings,labels=names(NPB.data),cex=.7)

```

##### Cronbach's alpha

```{r}

alpha(NPB.data[,c("NPB_1", "NPB_4", "NPB_5", "NPB_6")])

alpha(NPB.data[,c("NPB_2", "NPB_3", "NPB_7_i")])

```

#### 1 factor

```{r}

NPB.fa.1 <- factanal(NPB.data, factors = 1, rotation = "varimax")

print(NPB.fa.1, digits=2, sort=FALSE)

```

##### Cronbach's alpha

```{r}

alpha(NPB.data[,c("NPB_1", "NPB_2", "NPB_3", "NPB_4", "NPB_5", "NPB_6", "NPB_7_i")])

```

**Path Analyses for Aims Two and Three (Stata)**

cd "C:\ Insert Path to File Here \COVID misinformation"

* Readin dataset

import delimited "Data\Cleaned\COVID Misinformation clean wide.csv"

gen profile1 = profiles == 1

gen profile2 = profiles == 2

gen profile3 = profiles == 3

* Covariates:

* age, gender, race, vaccination, religious, political, severity, selfefficacy, fambehav, diagnose, infect, and trust(pre)

*************

*** Aim 2 ***

*************

** Multinomial model

gsem (arm trust2 age i.gender i.race i.vaccination religious political severity selfefficacy fambehav i.diagnose i.infect trust1 -> profiles, family(multinomial) link(logit)) (arm age i.gender i.race i.vaccination religious political severity selfefficacy fambehav i.diagnose i.infect trust1 -> trust2, ), nocapslatent

estat ic

nlcom (DirectEffect_2vs1: _b[2.profiles:arm]) (IndirectEffect_2vs1: _b[2.profiles:trust2]*_b[trust2:arm]) (TotalEffect_2vs1: _b[2.profiles:arm]+_b[2.profiles:trust2]*_b[trust2:arm]) (DirectEffect_3vs1: _b[3.profiles:arm]) (IndirectEffect_3vs1: _b[3.profiles:trust2]*_b[trust2:arm]) (TotalEffect_3vs1: _b[3.profiles:arm]+_b[3.profiles:trust2]*_b[trust2:arm])

nlcom (DirectEffectOR_2vs1: exp(_b[2.profiles:arm])) (IndirectEffectOR_2vs1: exp(_b[2.profiles:trust2]*_b[trust2:arm])) (TotalEffectOR_2vs1: exp(_b[2.profiles:arm]+_b[2.profiles:trust2]*_b[trust2:arm])) (DirectEffectOR_3vs1: exp(_b[3.profiles:arm])) (IndirectEffectOR_3vs1: exp(_b[3.profiles:trust2]*_b[trust2:arm])) (TotalEffectOR_3vs1: exp(_b[3.profiles:arm]+_b[3.profiles:trust2]*_b[trust2:arm]))

** Binomial model

gsem (arm trust2 age i.gender i.race i.vaccination religious political severity selfefficacy fambehav i.diagnose i.infect trust1 -> profile1, family(binomial) link(logit)) (arm age i.gender i.race i.vaccination religious political severity selfefficacy fambehav i.diagnose i.infect trust1 -> trust2, ), nocapslatent

estat ic

nlcom (DirectEffect: _b[profile1:arm]) (IndirectEffect: _b[profile1:trust2]*_b[trust2:arm]) (TotalEffect: _b[profile1:arm]+_b[profile1:trust2]*_b[trust2:arm])

nlcom (DirectEffectOR: exp(_b[profile1:arm])) (IndirectEffectOR: exp(_b[profile1:trust2]*_b[trust2:arm])) (TotalEffectOR: exp(_b[profile1:arm]+_b[profile1:trust2]*_b[trust2:arm]))

gsem (arm trust2 age i.gender i.race i.vaccination religious political severity selfefficacy fambehav i.diagnose i.infect trust1 -> profile2, family(binomial) link(logit)) (arm age i.gender i.race i.vaccination religious political severity selfefficacy fambehav i.diagnose i.infect trust1 -> trust2, ), nocapslatent

estat ic

nlcom (DirectEffect: _b[profile2:arm]) (IndirectEffect: _b[profile2:trust2]*_b[trust2:arm]) (TotalEffect: _b[profile2:arm]+_b[profile2:trust2]*_b[trust2:arm])

nlcom (DirectEffectOR: exp(_b[profile2:arm])) (IndirectEffectOR: exp(_b[profile2:trust2]*_b[trust2:arm])) (TotalEffectOR: exp(_b[profile2:arm]+_b[profile2:trust2]*_b[trust2:arm]))

gsem (arm trust2 age i.gender i.race i.vaccination religious political severity selfefficacy fambehav i.diagnose i.infect trust1 -> profile3, family(binomial) link(logit)) (arm age i.gender i.race i.vaccination religious political severity selfefficacy fambehav i.diagnose i.infect trust1 -> trust2, ), nocapslatent

estat ic

nlcom (DirectEffect: _b[profile3:arm]) (IndirectEffect: _b[profile3:trust2]*_b[trust2:arm]) (TotalEffect: _b[profile3:arm]+_b[profile3:trust2]*_b[trust2:arm])

nlcom (DirectEffectOR: exp(_b[profile3:arm])) (IndirectEffectOR: exp(_b[profile3:trust2]*_b[trust2:arm])) (TotalEffectOR: exp(_b[profile3:arm]+_b[profile3:trust2]*_b[trust2:arm]))

********************************************************************

*** Aim 3 Using original item 7 (NPB_7) - Trust in science as IV ***

********************************************************************

* NPB as one factor

** Multinomial model

gsem (trust2 i.profiles age i.gender i.race i.vaccination religious political severity selfefficacy fambehav i.diagnose i.infect trust1 -> npb, ) (trust2 age i.gender i.race i.vaccination religious political severity selfefficacy fambehav i.diagnose i.infect trust1 -> profiles, family(multinomial) link(logit)), nocapslatent

estat ic

nlcom (DirectEffect_2vs1: _b[npb:trust2]) (IndirectEffect_2vs1: _b[npb:2.profiles]*_b[2.profiles:trust2]) (TotalEffect_2vs1: _b[npb:trust2]+_b[npb:2.profiles]*_b[2.profiles:trust2]) (DirectEffect_3vs1: _b[npb:trust2]) (IndirectEffect_3vs1: _b[npb:3.profiles]*_b[3.profiles:trust2]) (TotalEffect_3vs1: _b[npb:trust2]+_b[npb:3.profiles]*_b[3.profiles:trust2])

** Binomial model

gsem (trust2 profile1 age i.gender i.race i.vaccination religious political severity selfefficacy fambehav i.diagnose i.infect trust1 -> npb, ) (trust2 age i.gender i.race i.vaccination religious political severity selfefficacy fambehav i.diagnose i.infect trust1 -> profile1, family(binomial) link(logit)), nocapslatent

estat ic

nlcom (DirectEffect: _b[npb:trust2]) (IndirectEffect: _b[npb:profile1]*_b[profile1:trust2]) (TotalEffect: _b[npb:trust2]+_b[npb:profile1]*_b[profile1:trust2])

gsem (trust2 profile2 age i.gender i.race i.vaccination religious political severity selfefficacy fambehav i.diagnose i.infect trust1 -> npb, ) (trust2 age i.gender i.race i.vaccination religious political severity selfefficacy fambehav i.diagnose i.infect trust1 -> profile2, family(binomial) link(logit)), nocapslatent

estat ic

nlcom (DirectEffect: _b[npb:trust2]) (IndirectEffect: _b[npb:profile2]*_b[profile2:trust2]) (TotalEffect: _b[npb:trust2]+_b[npb:profile2]*_b[profile2:trust2])

gsem (trust2 profile3 age i.gender i.race i.vaccination religious political severity selfefficacy fambehav i.diagnose i.infect trust1 -> npb, ) (trust2 age i.gender i.race i.vaccination religious political severity selfefficacy fambehav i.diagnose i.infect trust1 -> profile3, family(binomial) link(logit)), nocapslatent

estat ic

nlcom (DirectEffect: _b[npb:trust2]) (IndirectEffect: _b[npb:profile3]*_b[profile3:trust2]) (TotalEffect: _b[npb:trust2]+_b[npb:profile3]*_b[profile3:trust2])

* NPB as two factors

** Multinomial model

gsem (trust2 i.profiles age i.gender i.race i.vaccination religious political severity selfefficacy fambehav i.diagnose i.infect trust1 -> npb_f1, ) (trust2 age i.gender i.race i.vaccination religious political severity selfefficacy fambehav i.diagnose i.infect trust1 -> profiles, family(multinomial) link(logit)), nocapslatent

estat ic

nlcom (DirectEffect_2vs1: _b[npb_f1:trust2]) (IndirectEffect_2vs1: _b[npb_f1:2.profiles]*_b[2.profiles:trust2]) (TotalEffect_2vs1: _b[npb_f1:trust2]+_b[npb_f1:2.profiles]*_b[2.profiles:trust2]) (DirectEffect_3vs1: _b[npb_f1:trust2]) (IndirectEffect_3vs1: _b[npb_f1:3.profiles]*_b[3.profiles:trust2]) (TotalEffect_3vs1: _b[npb_f1:trust2]+_b[npb_f1:3.profiles]*_b[3.profiles:trust2])

gsem (trust2 i.profiles age i.gender i.race i.vaccination religious political severity selfefficacy fambehav i.diagnose i.infect trust1 -> npb_f2, ) (trust2 age i.gender i.race i.vaccination religious political severity selfefficacy fambehav i.diagnose i.infect trust1 -> profiles, family(multinomial) link(logit)), nocapslatent

estat ic

nlcom (DirectEffect_2vs1: _b[npb_f2:trust2]) (IndirectEffect_2vs1: _b[npb_f2:2.profiles]*_b[2.profiles:trust2]) (TotalEffect_2vs1: _b[npb_f2:trust2]+_b[npb_f2:2.profiles]*_b[2.profiles:trust2]) (DirectEffect_3vs1: _b[npb_f2:trust2]) (IndirectEffect_3vs1: _b[npb_f2:3.profiles]*_b[3.profiles:trust2]) (TotalEffect_3vs1: _b[npb_f2:trust2]+_b[npb_f2:3.profiles]*_b[3.profiles:trust2])

** Binomial model

gsem (trust2 profile1 age i.gender i.race i.vaccination religious political severity selfefficacy fambehav i.diagnose i.infect trust1 -> npb_f1, ) (trust2 age i.gender i.race i.vaccination religious political severity selfefficacy fambehav i.diagnose i.infect trust1 -> profile1, family(binomial) link(logit)), nocapslatent

estat ic

nlcom (DirectEffect: _b[npb_f1:trust2]) (IndirectEffect: _b[npb_f1:profile1]*_b[profile1:trust2]) (TotalEffect: _b[npb_f1:trust2]+_b[npb_f1:profile1]*_b[profile1:trust2])

gsem (trust2 profile2 age i.gender i.race i.vaccination religious political severity selfefficacy fambehav i.diagnose i.infect trust1 -> npb_f1, ) (trust2 age i.gender i.race i.vaccination religious political severity selfefficacy fambehav i.diagnose i.infect trust1 -> profile2, family(binomial) link(logit)), nocapslatent

estat ic

nlcom (DirectEffect: _b[npb_f1:trust2]) (IndirectEffect: _b[npb_f1:profile2]*_b[profile2:trust2]) (TotalEffect: _b[npb_f1:trust2]+_b[npb_f1:profile2]*_b[profile2:trust2])

gsem (trust2 profile3 age i.gender i.race i.vaccination religious political severity selfefficacy fambehav i.diagnose i.infect trust1 -> npb_f1, ) (trust2 age i.gender i.race i.vaccination religious political severity selfefficacy fambehav i.diagnose i.infect trust1 -> profile3, family(binomial) link(logit)), nocapslatent

estat ic

nlcom (DirectEffect: _b[npb_f1:trust2]) (IndirectEffect: _b[npb_f1:profile3]*_b[profile3:trust2]) (TotalEffect: _b[npb_f1:trust2]+_b[npb_f1:profile3]*_b[profile3:trust2])

gsem (trust2 profile1 age i.gender i.race i.vaccination religious political severity selfefficacy fambehav i.diagnose i.infect trust1 -> npb_f2, ) (trust2 age i.gender i.race i.vaccination religious political severity selfefficacy fambehav i.diagnose i.infect trust1 -> profile1, family(binomial) link(logit)), nocapslatent

estat ic

nlcom (DirectEffect: _b[npb_f2:trust2]) (IndirectEffect: _b[npb_f2:profile1]*_b[profile1:trust2]) (TotalEffect: _b[npb_f2:trust2]+_b[npb_f2:profile1]*_b[profile1:trust2])

gsem (trust2 profile2 age i.gender i.race i.vaccination religious political severity selfefficacy fambehav i.diagnose i.infect trust1 -> npb_f2, ) (trust2 age i.gender i.race i.vaccination religious political severity selfefficacy fambehav i.diagnose i.infect trust1 -> profile2, family(binomial) link(logit)), nocapslatent

estat ic

nlcom (DirectEffect: _b[npb_f2:trust2]) (IndirectEffect: _b[npb_f2:profile2]*_b[profile2:trust2]) (TotalEffect: _b[npb_f2:trust2]+_b[npb_f2:profile2]*_b[profile2:trust2])

gsem (trust2 profile3 age i.gender i.race i.vaccination religious political severity selfefficacy fambehav i.diagnose i.infect trust1 -> npb_f2, ) (trust2 age i.gender i.race i.vaccination religious political severity selfefficacy fambehav i.diagnose i.infect trust1 -> profile3, family(binomial) link(logit)), nocapslatent

estat ic

nlcom (DirectEffect: _b[npb_f2:trust2]) (IndirectEffect: _b[npb_f2:profile3]*_b[profile3:trust2]) (TotalEffect: _b[npb_f2:trust2]+_b[npb_f2:profile3]*_b[profile3:trust2])

*********************************************************************

*** Aim 3 Using imputed item 7 (NPB_7_i) - Trust in science as IV ***

*********************************************************************

* NPB_i as one factor

** Multinomial model

gsem (trust2 i.profiles age i.gender i.race i.vaccination religious political severity selfefficacy fambehav i.diagnose i.infect trust1 -> npb_i, ) (trust2 age i.gender i.race i.vaccination religious political severity selfefficacy fambehav i.diagnose i.infect trust1 -> profiles, family(multinomial) link(logit)), nocapslatent

estat ic

nlcom (DirectEffect_2vs1: _b[npb_i:trust2]) (IndirectEffect_2vs1: _b[npb_i:2.profiles]*_b[2.profiles:trust2]) (TotalEffect_2vs1: _b[npb_i:trust2]+_b[npb_i:2.profiles]*_b[2.profiles:trust2]) (DirectEffect_3vs1: _b[npb_i:trust2]) (IndirectEffect_3vs1: _b[npb_i:3.profiles]*_b[3.profiles:trust2]) (TotalEffect_3vs1: _b[npb_i:trust2]+_b[npb_i:3.profiles]*_b[3.profiles:trust2])

** Binomial model

gsem (trust2 profile1 age i.gender i.race i.vaccination religious political severity selfefficacy fambehav i.diagnose i.infect trust1 -> npb_i, ) (trust2 age i.gender i.race i.vaccination religious political severity selfefficacy fambehav i.diagnose i.infect trust1 -> profile1, family(binomial) link(logit)), nocapslatent

estat ic

nlcom (DirectEffect: _b[npb_i:trust2]) (IndirectEffect: _b[npb_i:profile1]*_b[profile1:trust2]) (TotalEffect: _b[npb_i:trust2]+_b[npb_i:profile1]*_b[profile1:trust2])

gsem (trust2 profile2 age i.gender i.race i.vaccination religious political severity selfefficacy fambehav i.diagnose i.infect trust1 -> npb_i, ) (trust2 age i.gender i.race i.vaccination religious political severity selfefficacy fambehav i.diagnose i.infect trust1 -> profile2, family(binomial) link(logit)), nocapslatent

estat ic

nlcom (DirectEffect: _b[npb_i:trust2]) (IndirectEffect: _b[npb_i:profile2]*_b[profile2:trust2]) (TotalEffect: _b[npb_i:trust2]+_b[npb_i:profile2]*_b[profile2:trust2])

gsem (trust2 profile3 age i.gender i.race i.vaccination religious political severity selfefficacy fambehav i.diagnose i.infect trust1 -> npb_i, ) (trust2 age i.gender i.race i.vaccination religious political severity selfefficacy fambehav i.diagnose i.infect trust1 -> profile3, family(binomial) link(logit)), nocapslatent

estat ic

nlcom (DirectEffect: _b[npb_i:trust2]) (IndirectEffect: _b[npb_i:profile3]*_b[profile3:trust2]) (TotalEffect: _b[npb_i:trust2]+_b[npb_i:profile3]*_b[profile3:trust2])

* NPB as two factors

** Multinomial model

gsem (trust2 i.profiles age i.gender i.race i.vaccination religious political severity selfefficacy fambehav i.diagnose i.infect trust1 -> npb_f1_i, ) (trust2 age i.gender i.race i.vaccination religious political severity selfefficacy fambehav i.diagnose i.infect trust1 -> profiles, family(multinomial) link(logit)), nocapslatent

estat ic

nlcom (DirectEffect_2vs1: _b[npb_f1_i:trust2]) (IndirectEffect_2vs1: _b[npb_f1_i:2.profiles]*_b[2.profiles:trust2]) (TotalEffect_2vs1: _b[npb_f1_i:trust2]+_b[npb_f1_i:2.profiles]*_b[2.profiles:trust2]) (DirectEffect_3vs1: _b[npb_f1_i:trust2]) (IndirectEffect_3vs1: _b[npb_f1_i:3.profiles]*_b[3.profiles:trust2]) (TotalEffect_3vs1: _b[npb_f1_i:trust2]+_b[npb_f1_i:3.profiles]*_b[3.profiles:trust2])

gsem (trust2 i.profiles age i.gender i.race i.vaccination religious political severity selfefficacy fambehav i.diagnose i.infect trust1 -> npb_f2_i, ) (trust2 age i.gender i.race i.vaccination religious political severity selfefficacy fambehav i.diagnose i.infect trust1 -> profiles, family(multinomial) link(logit)), nocapslatent

estat ic

nlcom (DirectEffect_2vs1: _b[npb_f2_i:trust2]) (IndirectEffect_2vs1: _b[npb_f2_i:2.profiles]*_b[2.profiles:trust2]) (TotalEffect_2vs1: _b[npb_f2_i:trust2]+_b[npb_f2_i:2.profiles]*_b[2.profiles:trust2]) (DirectEffect_3vs1: _b[npb_f2_i:trust2]) (IndirectEffect_3vs1: _b[npb_f2_i:3.profiles]*_b[3.profiles:trust2]) (TotalEffect_3vs1: _b[npb_f2_i:trust2]+_b[npb_f2_i:3.profiles]*_b[3.profiles:trust2])

** Binomial model

gsem (trust2 profile1 age i.gender i.race i.vaccination religious political severity selfefficacy fambehav i.diagnose i.infect trust1 -> npb_f1_i, ) (trust2 age i.gender i.race i.vaccination religious political severity selfefficacy fambehav i.diagnose i.infect trust1 -> profile1, family(binomial) link(logit)), nocapslatent

estat ic

nlcom (DirectEffect: _b[npb_f1_i:trust2]) (IndirectEffect: _b[npb_f1_i:profile1]*_b[profile1:trust2]) (TotalEffect: _b[npb_f1_i:trust2]+_b[npb_f1_i:profile1]*_b[profile1:trust2])

gsem (trust2 profile2 age i.gender i.race i.vaccination religious political severity selfefficacy fambehav i.diagnose i.infect trust1 -> npb_f1_i, ) (trust2 age i.gender i.race i.vaccination religious political severity selfefficacy fambehav i.diagnose i.infect trust1 -> profile2, family(binomial) link(logit)), nocapslatent

estat ic

nlcom (DirectEffect: _b[npb_f1_i:trust2]) (IndirectEffect: _b[npb_f1_i:profile2]*_b[profile2:trust2]) (TotalEffect: _b[npb_f1_i:trust2]+_b[npb_f1_i:profile2]*_b[profile2:trust2])

gsem (trust2 profile3 age i.gender i.race i.vaccination religious political severity selfefficacy fambehav i.diagnose i.infect trust1 -> npb_f1_i, ) (trust2 age i.gender i.race i.vaccination religious political severity selfefficacy fambehav i.diagnose i.infect trust1 -> profile3, family(binomial) link(logit)), nocapslatent

estat ic

nlcom (DirectEffect: _b[npb_f1_i:trust2]) (IndirectEffect: _b[npb_f1_i:profile3]*_b[profile3:trust2]) (TotalEffect: _b[npb_f1_i:trust2]+_b[npb_f1_i:profile3]*_b[profile3:trust2])

gsem (trust2 profile1 age i.gender i.race i.vaccination religious political severity selfefficacy fambehav i.diagnose i.infect trust1 -> npb_f2_i, ) (trust2 age i.gender i.race i.vaccination religious political severity selfefficacy fambehav i.diagnose i.infect trust1 -> profile1, family(binomial) link(logit)), nocapslatent

estat ic

nlcom (DirectEffect: _b[npb_f2_i:trust2]) (IndirectEffect: _b[npb_f2_i:profile1]*_b[profile1:trust2]) (TotalEffect: _b[npb_f2_i:trust2]+_b[npb_f2_i:profile1]*_b[profile1:trust2])

gsem (trust2 profile2 age i.gender i.race i.vaccination religious political severity selfefficacy fambehav i.diagnose i.infect trust1 -> npb_f2_i, ) (trust2 age i.gender i.race i.vaccination religious political severity selfefficacy fambehav i.diagnose i.infect trust1 -> profile2, family(binomial) link(logit)), nocapslatent

estat ic

nlcom (DirectEffect: _b[npb_f2_i:trust2]) (IndirectEffect: _b[npb_f2_i:profile2]*_b[profile2:trust2]) (TotalEffect: _b[npb_f2_i:trust2]+_b[npb_f2_i:profile2]*_b[profile2:trust2])

gsem (trust2 profile3 age i.gender i.race i.vaccination religious political severity selfefficacy fambehav i.diagnose i.infect trust1 -> npb_f2_i, ) (trust2 age i.gender i.race i.vaccination religious political severity selfefficacy fambehav i.diagnose i.infect trust1 -> profile3, family(binomial) link(logit)), nocapslatent

estat ic

nlcom (DirectEffect: _b[npb_f2_i:trust2]) (IndirectEffect: _b[npb_f2_i:profile3]*_b[profile3:trust2]) (TotalEffect: _b[npb_f2_i:trust2]+_b[npb_f2_i:profile3]*_b[profile3:trust2])

*******************************************************

*** Aim 3 Using original item 7 (NPB_7) - Arm as IV ***

*******************************************************

* NPB as one factor

** Multinomial model

gsem (arm i.profiles age i.gender i.race i.vaccination religious political severity selfefficacy fambehav i.diagnose i.infect trust1 -> npb, ) (arm age i.gender i.race i.vaccination religious political severity selfefficacy fambehav i.diagnose i.infect trust1 -> profiles, family(multinomial) link(logit)), nocapslatent

estat ic

nlcom (DirectEffect_2vs1: _b[npb:arm]) (IndirectEffect_2vs1: _b[npb:2.profiles]*_b[2.profiles:arm]) (TotalEffect_2vs1: _b[npb:arm]+_b[npb:2.profiles]*_b[2.profiles:arm]) (DirectEffect_3vs1: _b[npb:arm]) (IndirectEffect_3vs1: _b[npb:3.profiles]*_b[3.profiles:arm]) (TotalEffect_3vs1: _b[npb:arm]+_b[npb:3.profiles]*_b[3.profiles:arm])

** Binomial model

gsem (arm profile1 age i.gender i.race i.vaccination religious political severity selfefficacy fambehav i.diagnose i.infect trust1 -> npb, ) (arm age i.gender i.race i.vaccination religious political severity selfefficacy fambehav i.diagnose i.infect trust1 -> profile1, family(binomial) link(logit)), nocapslatent

estat ic

nlcom (DirectEffect: _b[npb:arm]) (IndirectEffect: _b[npb:profile1]*_b[profile1:arm]) (TotalEffect: _b[npb:arm]+_b[npb:profile1]*_b[profile1:arm])

gsem (arm profile2 age i.gender i.race i.vaccination religious political severity selfefficacy fambehav i.diagnose i.infect trust1 -> npb, ) (arm age i.gender i.race i.vaccination religious political severity selfefficacy fambehav i.diagnose i.infect trust1 -> profile2, family(binomial) link(logit)), nocapslatent

estat ic

nlcom (DirectEffect: _b[npb:arm]) (IndirectEffect: _b[npb:profile2]*_b[profile2:arm]) (TotalEffect: _b[npb:arm]+_b[npb:profile2]*_b[profile2:arm])

gsem (arm profile3 age i.gender i.race i.vaccination religious political severity selfefficacy fambehav i.diagnose i.infect trust1 -> npb, ) (arm age i.gender i.race i.vaccination religious political severity selfefficacy fambehav i.diagnose i.infect trust1 -> profile3, family(binomial) link(logit)), nocapslatent

estat ic

nlcom (DirectEffect: _b[npb:arm]) (IndirectEffect: _b[npb:profile3]*_b[profile3:arm]) (TotalEffect: _b[npb:arm]+_b[npb:profile3]*_b[profile3:arm])

* NPB as two factors

** Multinomial model

gsem (arm i.profiles age i.gender i.race i.vaccination religious political severity selfefficacy fambehav i.diagnose i.infect trust1 -> npb_f1, ) (arm age i.gender i.race i.vaccination religious political severity selfefficacy fambehav i.diagnose i.infect trust1 -> profiles, family(multinomial) link(logit)), nocapslatent

estat ic

nlcom (DirectEffect_2vs1: _b[npb_f1:arm]) (IndirectEffect_2vs1: _b[npb_f1:2.profiles]*_b[2.profiles:arm]) (TotalEffect_2vs1: _b[npb_f1:arm]+_b[npb_f1:2.profiles]*_b[2.profiles:arm]) (DirectEffect_3vs1: _b[npb_f1:arm]) (IndirectEffect_3vs1: _b[npb_f1:3.profiles]*_b[3.profiles:arm]) (TotalEffect_3vs1: _b[npb_f1:arm]+_b[npb_f1:3.profiles]*_b[3.profiles:arm])

gsem (arm i.profiles age i.gender i.race i.vaccination religious political severity selfefficacy fambehav i.diagnose i.infect trust1 -> npb_f2, ) (arm age i.gender i.race i.vaccination religious political severity selfefficacy fambehav i.diagnose i.infect trust1 -> profiles, family(multinomial) link(logit)), nocapslatent

estat ic

nlcom (DirectEffect_2vs1: _b[npb_f2:arm]) (IndirectEffect_2vs1: _b[npb_f2:2.profiles]*_b[2.profiles:arm]) (TotalEffect_2vs1: _b[npb_f2:arm]+_b[npb_f2:2.profiles]*_b[2.profiles:arm]) (DirectEffect_3vs1: _b[npb_f2:arm]) (IndirectEffect_3vs1: _b[npb_f2:3.profiles]*_b[3.profiles:arm]) (TotalEffect_3vs1: _b[npb_f2:arm]+_b[npb_f2:3.profiles]*_b[3.profiles:arm])

** Binomial model

gsem (arm profile1 age i.gender i.race i.vaccination religious political severity selfefficacy fambehav i.diagnose i.infect trust1 -> npb_f1, ) (arm age i.gender i.race i.vaccination religious political severity selfefficacy fambehav i.diagnose i.infect trust1 -> profile1, family(binomial) link(logit)), nocapslatent

estat ic

nlcom (DirectEffect: _b[npb_f1:arm]) (IndirectEffect: _b[npb_f1:profile1]*_b[profile1:arm]) (TotalEffect: _b[npb_f1:arm]+_b[npb_f1:profile1]*_b[profile1:arm])

gsem (arm profile2 age i.gender i.race i.vaccination religious political severity selfefficacy fambehav i.diagnose i.infect trust1 -> npb_f1, ) (arm age i.gender i.race i.vaccination religious political severity selfefficacy fambehav i.diagnose i.infect trust1 -> profile2, family(binomial) link(logit)), nocapslatent

estat ic

nlcom (DirectEffect: _b[npb_f1:arm]) (IndirectEffect: _b[npb_f1:profile2]*_b[profile2:arm]) (TotalEffect: _b[npb_f1:arm]+_b[npb_f1:profile2]*_b[profile2:arm])

gsem (arm profile3 age i.gender i.race i.vaccination religious political severity selfefficacy fambehav i.diagnose i.infect trust1 -> npb_f1, ) (arm age i.gender i.race i.vaccination religious political severity selfefficacy fambehav i.diagnose i.infect trust1 -> profile3, family(binomial) link(logit)), nocapslatent

estat ic

nlcom (DirectEffect: _b[npb_f1:arm]) (IndirectEffect: _b[npb_f1:profile3]*_b[profile3:arm]) (TotalEffect: _b[npb_f1:arm]+_b[npb_f1:profile3]*_b[profile3:arm])

gsem (arm profile1 age i.gender i.race i.vaccination religious political severity selfefficacy fambehav i.diagnose i.infect trust1 -> npb_f2, ) (arm age i.gender i.race i.vaccination religious political severity selfefficacy fambehav i.diagnose i.infect trust1 -> profile1, family(binomial) link(logit)), nocapslatent

estat ic

nlcom (DirectEffect: _b[npb_f2:arm]) (IndirectEffect: _b[npb_f2:profile1]*_b[profile1:arm]) (TotalEffect: _b[npb_f2:arm]+_b[npb_f2:profile1]*_b[profile1:arm])

gsem (arm profile2 age i.gender i.race i.vaccination religious political severity selfefficacy fambehav i.diagnose i.infect trust1 -> npb_f2, ) (arm age i.gender i.race i.vaccination religious political severity selfefficacy fambehav i.diagnose i.infect trust1 -> profile2, family(binomial) link(logit)), nocapslatent

estat ic

nlcom (DirectEffect: _b[npb_f2:arm]) (IndirectEffect: _b[npb_f2:profile2]*_b[profile2:arm]) (TotalEffect: _b[npb_f2:arm]+_b[npb_f2:profile2]*_b[profile2:arm])

gsem (arm profile3 age i.gender i.race i.vaccination religious political severity selfefficacy fambehav i.diagnose i.infect trust1 -> npb_f2, ) (arm age i.gender i.race i.vaccination religious political severity selfefficacy fambehav i.diagnose i.infect trust1 -> profile3, family(binomial) link(logit)), nocapslatent

estat ic

nlcom (DirectEffect: _b[npb_f2:arm]) (IndirectEffect: _b[npb_f2:profile3]*_b[profile3:arm]) (TotalEffect: _b[npb_f2:arm]+_b[npb_f2:profile3]*_b[profile3:arm])

********************************************************

*** Aim 3 Using imputed item 7 (NPB_7_i) - Arm as IV ***

********************************************************

* NPB_i as one factor

** Multinomial model

gsem (arm i.profiles age i.gender i.race i.vaccination religious political severity selfefficacy fambehav i.diagnose i.infect trust1 -> npb_i, ) (arm age i.gender i.race i.vaccination religious political severity selfefficacy fambehav i.diagnose i.infect trust1 -> profiles, family(multinomial) link(logit)), nocapslatent

estat ic

nlcom (DirectEffect_2vs1: _b[npb_i:arm]) (IndirectEffect_2vs1: _b[npb_i:2.profiles]*_b[2.profiles:arm]) (TotalEffect_2vs1: _b[npb_i:arm]+_b[npb_i:2.profiles]*_b[2.profiles:arm]) (DirectEffect_3vs1: _b[npb_i:arm]) (IndirectEffect_3vs1: _b[npb_i:3.profiles]*_b[3.profiles:arm]) (TotalEffect_3vs1: _b[npb_i:arm]+_b[npb_i:3.profiles]*_b[3.profiles:arm])

** Binomial model

gsem (arm profile1 age i.gender i.race i.vaccination religious political severity selfefficacy fambehav i.diagnose i.infect trust1 -> npb_i, ) (arm age i.gender i.race i.vaccination religious political severity selfefficacy fambehav i.diagnose i.infect trust1 -> profile1, family(binomial) link(logit)), nocapslatent

estat ic

nlcom (DirectEffect: _b[npb_i:arm]) (IndirectEffect: _b[npb_i:profile1]*_b[profile1:arm]) (TotalEffect: _b[npb_i:arm]+_b[npb_i:profile1]*_b[profile1:arm])

gsem (arm profile2 age i.gender i.race i.vaccination religious political severity selfefficacy fambehav i.diagnose i.infect trust1 -> npb_i, ) (arm age i.gender i.race i.vaccination religious political severity selfefficacy fambehav i.diagnose i.infect trust1 -> profile2, family(binomial) link(logit)), nocapslatent

estat ic

nlcom (DirectEffect: _b[npb_i:arm]) (IndirectEffect: _b[npb_i:profile2]*_b[profile2:arm]) (TotalEffect: _b[npb_i:arm]+_b[npb_i:profile2]*_b[profile2:arm])

gsem (arm profile3 age i.gender i.race i.vaccination religious political severity selfefficacy fambehav i.diagnose i.infect trust1 -> npb_i, ) (arm age i.gender i.race i.vaccination religious political severity selfefficacy fambehav i.diagnose i.infect trust1 -> profile3, family(binomial) link(logit)), nocapslatent

estat ic

nlcom (DirectEffect: _b[npb_i:arm]) (IndirectEffect: _b[npb_i:profile3]*_b[profile3:arm]) (TotalEffect: _b[npb_i:arm]+_b[npb_i:profile3]*_b[profile3:arm])

* NPB as two factors

** Multinomial model

gsem (arm i.profiles age i.gender i.race i.vaccination religious political severity selfefficacy fambehav i.diagnose i.infect trust1 -> npb_f1_i, ) (arm age i.gender i.race i.vaccination religious political severity selfefficacy fambehav i.diagnose i.infect trust1 -> profiles, family(multinomial) link(logit)), nocapslatent

estat ic

nlcom (DirectEffect_2vs1: _b[npb_f1_i:arm]) (IndirectEffect_2vs1: _b[npb_f1_i:2.profiles]*_b[2.profiles:arm]) (TotalEffect_2vs1: _b[npb_f1_i:arm]+_b[npb_f1_i:2.profiles]*_b[2.profiles:arm]) (DirectEffect_3vs1: _b[npb_f1_i:arm]) (IndirectEffect_3vs1: _b[npb_f1_i:3.profiles]*_b[3.profiles:arm]) (TotalEffect_3vs1: _b[npb_f1_i:arm]+_b[npb_f1_i:3.profiles]*_b[3.profiles:arm])

gsem (arm i.profiles age i.gender i.race i.vaccination religious political severity selfefficacy fambehav i.diagnose i.infect trust1 -> npb_f2_i, ) (arm age i.gender i.race i.vaccination religious political severity selfefficacy fambehav i.diagnose i.infect trust1 -> profiles, family(multinomial) link(logit)), nocapslatent

estat ic

nlcom (DirectEffect_2vs1: _b[npb_f2_i:arm]) (IndirectEffect_2vs1: _b[npb_f2_i:2.profiles]*_b[2.profiles:arm]) (TotalEffect_2vs1: _b[npb_f2_i:arm]+_b[npb_f2_i:2.profiles]*_b[2.profiles:arm]) (DirectEffect_3vs1: _b[npb_f2_i:arm]) (IndirectEffect_3vs1: _b[npb_f2_i:3.profiles]*_b[3.profiles:arm]) (TotalEffect_3vs1: _b[npb_f2_i:arm]+_b[npb_f2_i:3.profiles]*_b[3.profiles:arm])

** Binomial model

gsem (arm profile1 age i.gender i.race i.vaccination religious political severity selfefficacy fambehav i.diagnose i.infect trust1 -> npb_f1_i, ) (arm age i.gender i.race i.vaccination religious political severity selfefficacy fambehav i.diagnose i.infect trust1 -> profile1, family(binomial) link(logit)), nocapslatent

estat ic

nlcom (DirectEffect: _b[npb_f1_i:arm]) (IndirectEffect: _b[npb_f1_i:profile1]*_b[profile1:arm]) (TotalEffect: _b[npb_f1_i:arm]+_b[npb_f1_i:profile1]*_b[profile1:arm])

gsem (arm profile2 age i.gender i.race i.vaccination religious political severity selfefficacy fambehav i.diagnose i.infect trust1 -> npb_f1_i, ) (arm age i.gender i.race i.vaccination religious political severity selfefficacy fambehav i.diagnose i.infect trust1 -> profile2, family(binomial) link(logit)), nocapslatent

estat ic

nlcom (DirectEffect: _b[npb_f1_i:arm]) (IndirectEffect: _b[npb_f1_i:profile2]*_b[profile2:arm]) (TotalEffect: _b[npb_f1_i:arm]+_b[npb_f1_i:profile2]*_b[profile2:arm])

gsem (arm profile3 age i.gender i.race i.vaccination religious political severity selfefficacy fambehav i.diagnose i.infect trust1 -> npb_f1_i, ) (arm age i.gender i.race i.vaccination religious political severity selfefficacy fambehav i.diagnose i.infect trust1 -> profile3, family(binomial) link(logit)), nocapslatent

estat ic

nlcom (DirectEffect: _b[npb_f1_i:arm]) (IndirectEffect: _b[npb_f1_i:profile3]*_b[profile3:arm]) (TotalEffect: _b[npb_f1_i:arm]+_b[npb_f1_i:profile3]*_b[profile3:arm])

gsem (arm profile1 age i.gender i.race i.vaccination religious political severity selfefficacy fambehav i.diagnose i.infect trust1 -> npb_f2_i, ) (arm age i.gender i.race i.vaccination religious political severity selfefficacy fambehav i.diagnose i.infect trust1 -> profile1, family(binomial) link(logit)), nocapslatent

estat ic

nlcom (DirectEffect: _b[npb_f2_i:arm]) (IndirectEffect: _b[npb_f2_i:profile1]*_b[profile1:arm]) (TotalEffect: _b[npb_f2_i:arm]+_b[npb_f2_i:profile1]*_b[profile1:arm])

gsem (arm profile2 age i.gender i.race i.vaccination religious political severity selfefficacy fambehav i.diagnose i.infect trust1 -> npb_f2_i, ) (arm age i.gender i.race i.vaccination religious political severity selfefficacy fambehav i.diagnose i.infect trust1 -> profile2, family(binomial) link(logit)), nocapslatent

estat ic

nlcom (DirectEffect: _b[npb_f2_i:arm]) (IndirectEffect: _b[npb_f2_i:profile2]*_b[profile2:arm]) (TotalEffect: _b[npb_f2_i:arm]+_b[npb_f2_i:profile2]*_b[profile2:arm])

gsem (arm profile3 age i.gender i.race i.vaccination religious political severity selfefficacy fambehav i.diagnose i.infect trust1 -> npb_f2_i, ) (arm age i.gender i.race i.vaccination religious political severity selfefficacy fambehav i.diagnose i.infect trust1 -> profile3, family(binomial) link(logit)), nocapslatent

estat ic

nlcom (DirectEffect: _b[npb_f2_i:arm]) (IndirectEffect: _b[npb_f2_i:profile3]*_b[profile3:arm]) (TotalEffect: _b[npb_f2_i:arm]+_b[npb_f2_i:profile3]*_b[profile3:arm])

**Exploratory Regression Analysis within Aim Two (SAS)**

libname user 'C:\Insert Path to File Here\Full Study';

PROC IMPORT OUT= User.Aim2

DATAFILE= "C:\Insert Path to File Here\ClassProb.csv"

DBMS=CSV REPLACE;

GETNAMES=YES;

DATAROW=2;

RUN;

proc contents data=Aim2;

run;

Data CleanedAim2;

Set Aim2;

Rgender=gender;

Rrace=race;

RDiagnose=diagnose;

run;

**Suspect potentially strong associations between existing variables such as diagnosis and suspected infection. Given interest in perception the Infect variable may be better if they are highly related;

proc freq data=CleanedAim2;

Tables Rdiagnose*Infect / chisq;

run;

**This version of the model has some overspecification but it does not affect the findings (see next code block);

proc logistic data=CleanedAim2;

class arm (ref = "1") C (ref = "1") Rgender (ref = "1") Rrace (ref = "1") ethnicit (ref = "2") infect (ref = "2") / param = ref;

model C = Rgender Rrace ethnicit religiou politica severity selfeffi fambehav infect age posttrus arm / link=glogit;

run;

*********This is the exact same syntax as above but collapses small cells to avoid overspecification. This demonstrates that the results are functionally the same;

libname user 'C:\Insert Path to File Here\Full Study';

PROC IMPORT OUT= User.Aim2

DATAFILE= "C:\Insert Path to File Here\ClassProb.csv"

DBMS=CSV REPLACE;

GETNAMES=YES;

DATAROW=2;

RUN;

proc contents data=Aim2;

run;

proc freq data=Aim2;

Tables gender race ethnicit religiou politica severity selfeffi fambehav diagnose infect age posttrus arm;

run;

**Collapse gender, race, and diagnose variables

**Gender (exclude 10 who do not identify as male or female due to overspecification), Race nonWhite/Black/Asian collapsed into Other, Diagnose (unsure-->no);

Data CleanedAim2;

Set Aim2;

Rgender=gender;

If (gender=4) THEN rgender=.;

If (gender=3) THEN rgender=.;

Rrace=race;

If (race=3) THEN Rrace=6;

If (race=5) THEN Rrace=6;

RDiagnose=diagnose;

If (diagnose=3) THEN Rdiagnose=2;

run;

proc freq data=CleanedAim2;

Tables Rgender Rrace Rdiagnose;

run;

**Suspect potentially strong associations between existing variables such as diagnosis and suspected infection. Given interest in perception the Infect variable may be better if they are highly related;

proc freq data=CleanedAim2;

Tables Rdiagnose*Infect / chisq;

run;

**This version of the model is not overspecified, after removing the redundant variable and eliminating small categories for gender and race;

proc logistic data=CleanedAim2;

class arm (ref = "1") C (ref = "1") Rgender (ref = "1") Rrace (ref = "1") ethnicit (ref = "2") infect (ref = "2") / param = ref;

model C = Rgender Rrace ethnicit religiou politica severity selfeffi fambehav infect age posttrus arm / link=glogit;

run;

**Latent Profile Analysis for Aims Two and Three (Mplus)**

Data:

File is covidj.dat ;

Variable:

Names are

Gender Race Ethnicity Narratives_1 Narratives_2 Narratives_3 Narratives_4

Narratives_5 Narratives_6 Narratives_7 Religious Political Severity

SelfEfficacy FamBehav Diagnose Infect id Age PREtrust POSTTrust Arm;

Missing are all (-999) ;

Usevariables are

Narratives_1 Narratives_2 Narratives_3 Narratives_4

Narratives_5 Narratives_6 Narratives_7 ;

IDVARIABLE IS id;

CLASSES = c(3);

AUXILIARY = (R3STEP) Gender Race Ethnicity

Religious Political Severity

SelfEfficacy FamBehav Diagnose Infect

Age PREtrust POSTTrust Arm;

Analysis:

TYPE=MIXTURE ;

ESTIMATOR = MLR;

STARTS = 1000 250;

STITERATIONS = 500;

LRTSTARTS = 2 1 50 10;

MODEl:

%OVERALL%

[ Narratives_1 Narratives_2 Narratives_3 Narratives_4

Narratives_5 Narratives_6 Narratives_7];

Narratives_1 Narratives_2 Narratives_3 Narratives_4

Narratives_5 Narratives_6 Narratives_7 (Var1-Var7);

OUTPUT: TECH1 TECH4 TECH8 TECH11 TECH14;

SAVEDATA:

FILE IS COVIDRCT_3.dat;

SAVE = CPROBABILITIES;
